# Modeling fatty liver disease and progression with stem cell derived hepatocytes

**DOI:** 10.1038/s41598-025-34762-1

**Published:** 2026-01-07

**Authors:** Yao Wang, David Berlin, Yong Li, Lok Man Ko, Zhenzhu Qi, Jiayi Feng, Christopher T. Clark, Diandian Cheng, Melisa Andrade, Eric Potma, Quinton Smith

**Affiliations:** 1 Department of Chemical and Biomolecular Engineering, University of California, Irvine, Irvine, CA 92697 USA; 2 Sue and Bill Gross Stem Cell Research Center, University of California, Irvine, Irvine, CA 92697 USA; 3 Department of Chemistry, University of California, Irvine, Irvine, CA 92697 USA; 4 Department of Biomedical Engineering, University of California, Irvine, Irvine, CA 92697 USA; 5 Department of Materials Science and Engineering, University of California, Irvine, Irvine, CA 92697 USA

**Keywords:** Cell biology, Diseases, Gastroenterology, Stem cells

## Abstract

Metabolic dysfunction-associated steatotic liver disease (MASLD) is one of the most pervasive liver disorders. It can progress to metabolic dysfunction-associated steatohepatitis (MASH), hallmarked by increased inflammation and inclination to permanent liver damage. Given the limited treatment options available to patients, models that recapitulate critical features of disease pathogenesis are needed to improve drug development. Here, a MASLD model was developed by differentiating hepatocyte-like cells (HLCs) from human induced pluripotent stem cells (hiPSCs) in 2D and 3D, in which hepatic aggregates exhibited enhanced functionality. Induced HLCs exposed to free fatty acids led to a steatotic phenotype, partially reducing hepatic function. Biochemical cues released from induced HLCs promoted activation of fibroblasts, resulting in increased inflammatory cytokine secretion and extracellular matrix (ECM) deposition. These findings suggest paracrine signaling from hepatocytes to the surrounding stroma can trigger the progression of MASH and fibrosis. Collectively, our results demonstrate the utility of hiPSC derivatives for modeling liver diseases and reveal how heterotypic interactions can drive disease progression.

**Supplementary Information:**

The online version contains supplementary material available at 10.1038/s41598-025-34762-1.

## Introduction

The human liver is one of the major organs and performs more than five hundred functions, including metabolism, biosynthesis, and detoxification. The hierarchical repetition of functional units termed liver lobules makes up the entire liver, in which the hepatocyte is the most abundant cell type, regulating a majority of liver functions. When triggered by different mechanisms, liver diseases will lead to the eventual absence of liver functions^1^. Among various liver disorders, MASLD, formerly known as Non-alcoholic fatty liver disease (NAFLD) or MAFLD^2,3^ is one of the most common, affecting nearly 40% of adults worldwide^4^. The high correlation of MASLD to other health conditions, such as obesity and type 2 diabetes, suggests the potential cause of MASLD development, whereas the pathogenesis of MASLD has not been comprehensively revealed^5^. Recent genome-wide association studies (GWAS) show genes such as the patatin-like phospholipase domain-containing 3 (PNPLA3), which contain ancestral-specific single-nucleotide polymorphism (SNP), impact the risk for MASLD, unveiling the role of metabolic pathways in pathogenesis^6^. MASH, a more severe stage of MASLD with increased inflammation, is susceptible to fibrosis, thereby leaving permanent damage of liver scarring. The medical community has recommended weight loss at the MASLD stage to circumvent the progression to cirrhosis, yet there is no FDA-approved medication targeting MASLD. Of note, the FDA recently approved only one drug to target MASH^7^. Therefore, it is imperative that a model recapitulating critical features of MASLD must be developed for better elucidation of disease pathogenesis and evaluation of drug screening.

Current research models of MASLD mainly utilize immortalized hepatocytes^6–23^, primary human hepatocytes (PHHs)^9,24–30^, or animal models^6,31^. Still, drawbacks include partial functional loss, limited in vitro lifetime, or species variation, which fail to recapitulate human liver function accurately. Human induced pluripotent stem cell (hiPSC)-derived MASLD models depict a novel approach with promising progress^32–41^. Nevertheless, these modeling strategies have several limitations, including the inability to investigate the crosstalk among various organs^42^. Here, we developed a new MASLD model in both 2D and 3D that showcases steatosis and partial hepatic functional loss by inducing hiPSC-derived hepatocyte-like cells (HLC). Biochemical cues released from our MASLD model promote myofibroblast features, suggesting possible interactions that mediate the onset of MASH and progression to fibrosis.

## Results

### Stepwise differentiation of functional HLC from HiPSC

**Fig. 1:**
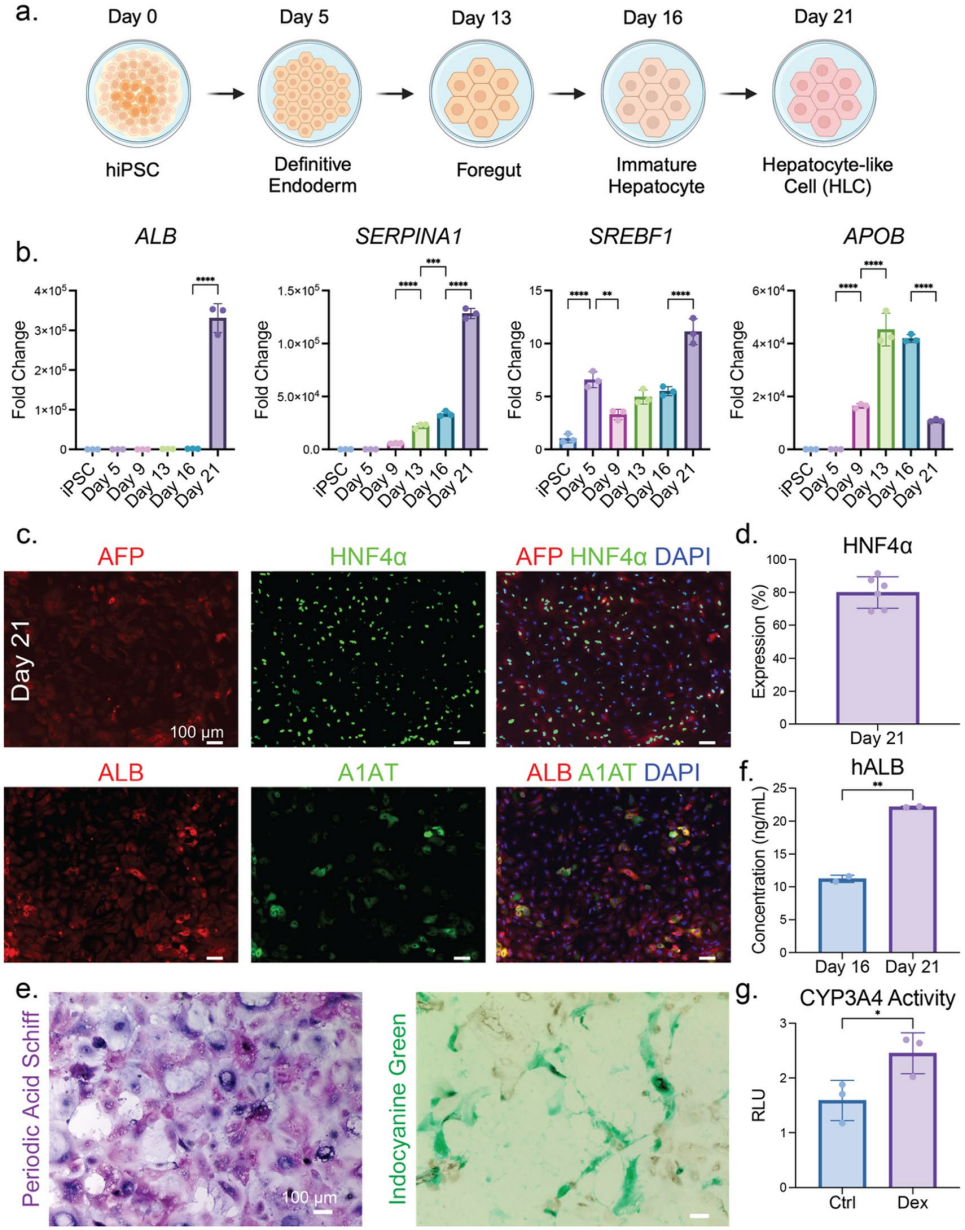
Directed differentiation of HLCs from hiPSCs. (**a**) Schematic of hiPSC differentiation protocol to HLCs. Crucial time points and developmental stages are specified. (**b**) qRT-PCR results of select hepatic markers at different time points of HLC derivation. Representative results from 3 biological replicates of differentiation, and each data point entails one technical replicate of the reaction. Unless indicated below, all *p*-values are less than 0.0001 (****) or larger than 0.9999 (without annotation) for every two adjacent comparisons. For *SERPINA1*, *p* = 0.0833 for Day 5 vs. Day 9, *p* = 0.0008 for Day 13 vs. Day 16. For *SREBF1*, *p* = 0.0012 for Day 5 vs. Day 9, *p* = 0.1227 for Day 9 vs. Day 13, *p* = 0.9263 for Day 13 vs. Day 16. For *APOB*, *p* = 0.6226 for Day 13 vs. Day 16. (**c**) Immunofluorescence images of select haptic markers in HLC (Day 21). Representative results of 3 biological replicates of differentiation. (**d**) HNF4α expression positivity, which is calculated by positive nuclear expression per nucleus. Representative results from 3 biological replicates of differentiation, and each data point entails one technical replicate of the enumerated cell. The p-value of the Shapiro-Wilk test is 0.4436. (**e**) Functional staining for HLC. PAS was used to visualize glycogen biosynthesis and storage, and ICG was used to test drug uptake. Representative results of 3 biological replicates of differentiation. (**f**) Human albumin ELISA result comparing albumin secretion from HLC (Day 21) to immature hepatocyte (Day 16), where *p* = 0.0013. Representative results of at least 4 biological replicates of differentiation, and each data point entails one technical replicate of the reaction. (**g**) CYP3A4 activity for HLC after treatment with dexamethasone, where *p* = 0.046. Representative results of 3 biological replicates of differentiation, and each data point entails one technical replicate of luminescence reading from one normalized CYP3A4 reaction. RLU, relative light unit.

Recent studies on hepatic differentiation from hiPSC have expanded the toolbox for understanding early human liver development, with the potential to be leveraged in preclinical settings for translational applications. Bearing the initial ambition to satisfy the shortage in liver transplants, hepatic differentiation from hiPSCs has been advanced for over a decade and has been exploited to generate and renew the supply of HLC to construct modeling systems for various liver diseases^43^. To this end, we first utilized a 2D differentiation approach modified from Dao Thi et al.^44^ administering a well-established cocktail of growth factors, including hepatocyte growth factor (HGF), dexamethasone, and oncostatin-M (OSM). After 21 days of differentiation, a monolayer of HLCs was generated and adopted to derive a MASLD model (Fig. 1a).

Media conditions were defined to induce a definitive endoderm signature first from hiPSCs. By day 5 of differentiation, qRT-PCR results showed an increase in Forkhead box protein A2 (*FoxA2*) expression along with dissipation of Sex determining region Y-box 2 (*SOX2*) (Fig. S1a). Then we tailored the growth factors to induce hepatic differentiation, and at the foregut to hepatoblast stage between day 9 to 13, we observed amplification in mRNA expression of hepatic markers, including Hepatocyte nuclear factor 4 alpha (*HNF4α*), Sterol regulatory element binding transcription factor 1 (*SREBF1*), and Apolipoprotein B (*APOB*). By day 21, a surge in the gene expression of Albumin (*ALB*) and Serpin family A member 1 (*SERPINA1*) demonstrated enrichment of hepatic identity. The expression of biliary marker Keratin 19 (*KRT19*) was upregulated by day 9 and was maintained till day 21 (Figs. 1b and S1b). Hepatic markers were also visualized by immunofluorescence, highlighting the expression of HNF4α, ALB, and Alpha-1 antitrypsin (A1AT, Fig. 1c). In particular, about 80% of the HLCs are positive with HNF4α staining (Fig. 1d). Biliary marker Sex determining region Y-box 9 (SOX9) and immature hepatocyte marker Alpha-fetoprotein (AFP) remained low by day 21 in comparison to day 16 (Fig. S1c). However, the hepatoblast marker epithelial cell adhesion molecule (EpCam) remained on the cell membrane by day 21, indicating the shortfall of HLC maturation compared to PHH (Fig. S1d)^45^. We also verified the expression of the aforementioned hepatocyte markers in the HLCs differentiated from another iPSC line (Fig. S2).

In addition to monitoring marker expression, we also investigated additional hepatocyte functionality of our derived HLCs. Periodic acid-Schiff (PAS) staining indicated the HLCs were capable of glycogen biosynthesis and storage, while Indocyanine Green (ICG) uptake indicated functional organic anion transporting capability (Fig. 1e). Secretion of albumin in the cell culture medium reached the apex by day 21, corroborating that the HLCs can perform hallmark hepatocyte functions (Fig. 1f). Detoxification is another crucial function of the liver, and a superfamily of enzymes called cytochrome P450 (CYP) is largely responsible for this function. We set out to detect one major member of the CYP superfamily and demonstrated increased CYP3A4 activity when HLCs were treated with drugs (Fig. 1g). Altogether, these results suggest that the differentiated HLCs demonstrated satisfactory hepatic maturity in terms of hepatic marker expression and functionality.

## Generation of HLC-derived hepatic aggregates

**Fig. 2:**
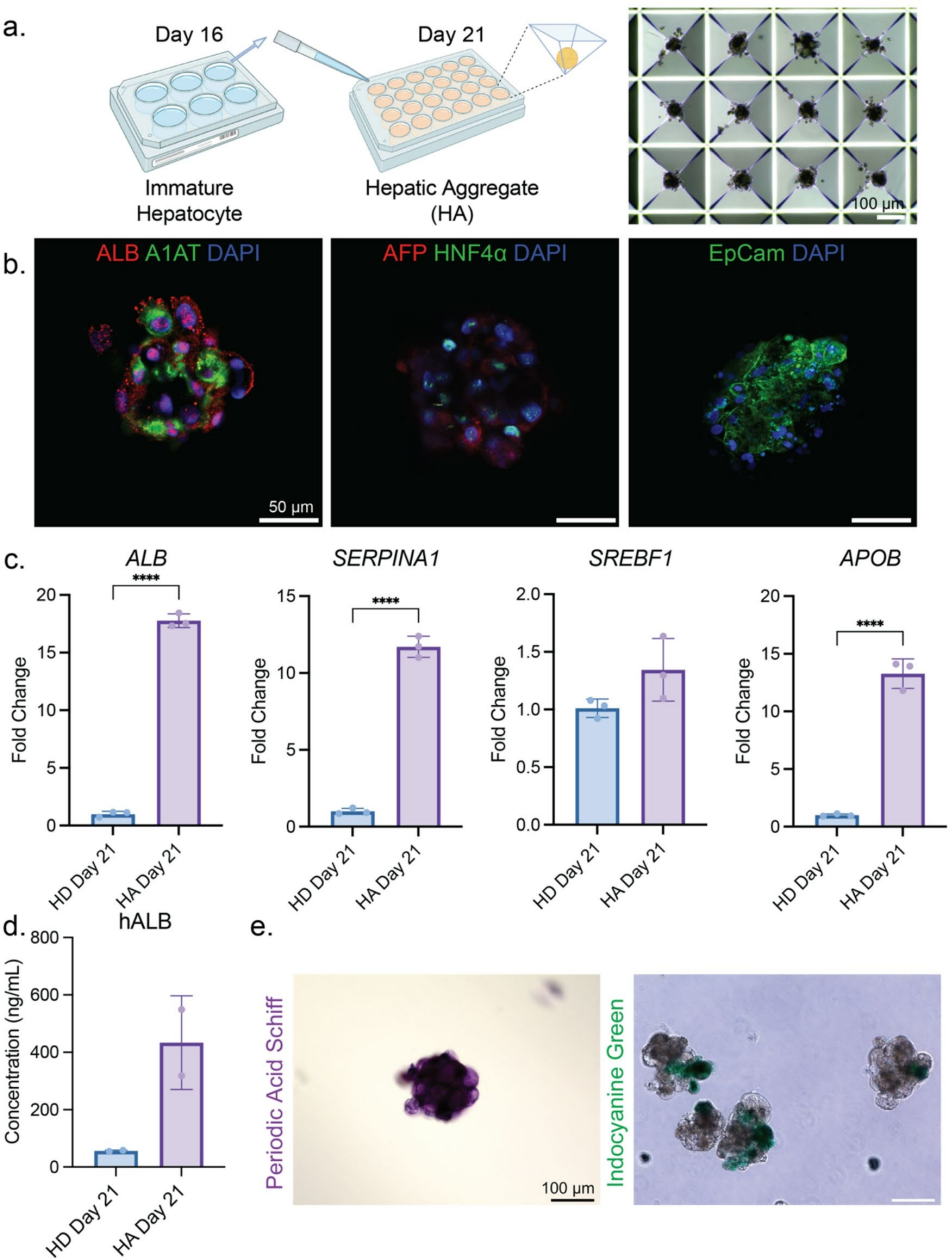
3D aggregate formation leads to enhanced hepatic function in HLCs. (**a**) Schematic of HA formation, along with the brightfield image showing HAs in Aggrewell molds. (**b**) Immunofluorescence images of HA stained with select hepatic markers. Representative results of 3 biological replicates of differentiation. (**c**) qRT-PCR results of select hepatic markers comparing the 2D and 3D HLC derivation. Representative results of 3 biological replicates of differentiation, and each data point entails one technical replicate of the reaction. The p-value for *SREBF1* is 0.1121 and is less than 0.0001 for the other genes. (**d**) Human albumin ELISA result comparing the secreted albumin from 2D and 3D HLC derivation, where *p* = 0.0821. Representative results of 3 biological replicates of differentiation, and each data point entails one technical replicate of the reaction. (**e**) Functional staining for HA. PAS was for glycogen biosynthesis and storage, and ICG was for drug uptake. Representative results from 3 biological replicates of differentiation.

Hepatocytes in vivo display a basolateral-apical polarity with their basolateral membranes facing the space of Disse, which interfaces with the sinusoidal blood, while the apical membranes form the bile canaliculi between adjacent hepatocytes^1^. HLCs derived from conventional 2D culture can hardly achieve this polarity, but it has been demonstrated through transwell, collagen sandwich, and micropatterned co-culture systems^44,46^. In addition, various engineering techniques have been employed to generate HLC in 3D, providing microenvironment cues that can support maturation^43^. Taking advantage of our 2D differentiation protocol, we generated hepatic aggregates (HAs) by seeding the cells at the hepatoblast stage into a pyramidal-shaped Aggrewell plate that encourages cell compaction (Fig. 2a). The generated HAs are of consistent sizes (Fig. S3c).

To characterize our HAs, we used immunofluorescence to confirm the presence of both immature and mature hepatic markers, including HNF4α, AFP, ALB, A1AT, and EpCam (Fig. 2b). We then compared the HAs to 2D-derived HLCs. From qRT-PCR results, we found a significant increase in the expression of *ALB*, *SERPINA1*, and *APOB*, suggesting improved functionality for 3D-derived hepatic aggregates (Fig. 2c). ELISA analysis revealed a moderate trend of augmented albumin secretion (Fig. 2d); however, there was no significant difference in urea metabolism between the HA and the 2D counterpart (Fig. S3a). PAS staining and ICG uptake were positive in HA, demonstrating that 3D culture sustains hepatic functionalities no less than traditional 2D hepatic differentiation approaches (Fig. 2e).

## MASLD induction attenuates hepatic functions

**Fig. 3:**
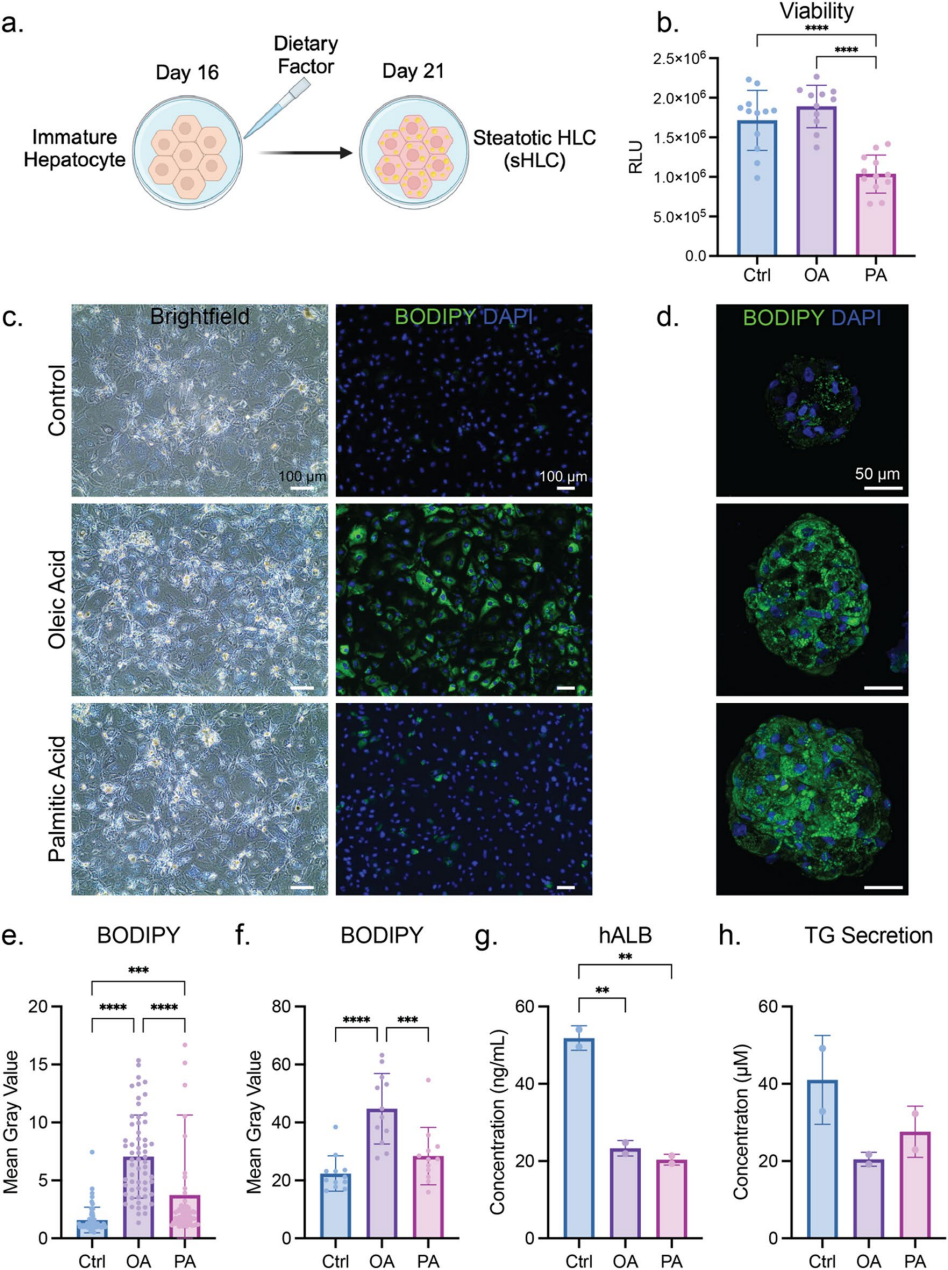
MASLD induction in hiPSC-derived HLCs. (**a**) Schematic of inducing a MASLD model during HLC maturation. (**b**) Cell viability of HLC and sHLCs induced by OA or PA. Representative results of 3 biological replicates, where *n* = 12 per condition. Each data point entails one technical replicate of the reaction. The *p*-value is 0.3585 for Ctrl vs. OA, and less than 0.0001 for Ctrl vs. PA and OA vs. PA. The p-values of the Shapiro-Wilk test are 0.2513, 0.5132, and 0.7657 for Ctrl, OA, and PA, respectively. (**c**) 2D brightfield and fluorescence images revealed a morphological change of sHLC in contrast to HLC. Representative results of 3 biological replicates of differentiation. (**d**) Fluorescence images of HA, OA-induced, and PA-induced HA. Representative results of 3 biological replicates of differentiation. The quantification of BODIPY in (**e**) 2D and (**f**) 3D was achieved by measuring the fluorescent intensity (mean gray value) per cell for 2D and per HA for 3D. Representative results of 3 biological replicates of differentiation. Each data point entails one technical replicate of the enumerated cell for 2D, and one technical replicate of the enumerated HA for 3D. For 2D, all conditions *n* = 60. The *p*-values of the Shapiro-Wilk test are < 0.0001, 0.0201, and < 0.0001 for Ctrl, OA, and PA, respectively. The Kruskal-Wallis test was performed for this comparison instead of one-way ANOVA. The *p*-value is < 0.0001 for Ctrl vs. OA, 0.0002 for Ctrl vs. PA, and < 0.0001 for OA vs. PA. For 3D, all conditions *n* = 12. The *p*-value is < 0.0001 for Ctrl vs. OA, 0.2953 for Ctrl vs. PA, and 0.0007 for OA vs. PA. The *p*-values of the Shapiro-Wilk test are 0.0137, 0.5944, and 0.0433 for Ctrl, OA, and PA, respectively. (**g**) Human albumin ELISA result comparing the secreted albumin from HLC and sHLCs induced by OA or PA. Representative results of 3 biological replicates of differentiation, and each data point entails one technical replicate of the reaction. The p-value is 0.0024 for Ctrl vs. OA, 0.0018 for Ctrl vs. PA, and 0.4972 for OA vs. PA. (**h**) TG secretion from HLC and sHLCs induced by OA or PA. Two technical replicates of the reaction were performed for 3 biological replicates of differentiation. Representative results of 3 biological replicates of differentiation. The *p*-value is 0.9926 for Ctrl vs. OA, 0.9943 for Ctrl vs. PA, and 0.9742 for OA vs. PA.

To model MASLD, a variety of dietary factors, including glucose, insulin, oleic acid (OA), palmitic acid (PA), and cholesterol, were supplemented during the maturation of HLCs (Fig. 3a). We tested a range of concentrations for each dietary factor to optimize the dosage give rise to the most steatotic response in the HLC. Based on BODIPY staining, we were able to evaluate the degree of lipid accumulation across all dietary factors (Fig. S4). Intracellular lipid accumulation was dose-dependent, yet for PA and cholesterol, high concentrations resulted in cell death^7,12^. We determined that administration of 100 µM OA was the optimal condition for MASLD induction, leading to the most significant steatosis phenotype with the most intense BODIPY fluorescence signal (Fig. 3e, f). After optimized free fatty acid treatment, steatotic HLC (sHLC) was generated, showing a distinct morphology compared to healthy HLC, where the sHLCs were teemed with lipid droplets (LDs, Fig. 3c). The optimized OA induction was also applied to HAs, where BODIPY intensity was higher for OA-induced HA than untreated controls (Fig. 3d). Similarly, the PA was optimized at 50 µM to induce lipid accumulation without causing extensive cell death, but the lipid content was less than those administered with OA in both 2D and 3D (Fig. 3c–f).

We then tested how the MASLD model mediated hepatic functions. We first confirmed that PA was detrimental to HLCs through a viability assay, as demonstrated by other groups (Fig. 3b)^48^. For some hepatic markers, including *HNF4α* and *SERPINA1*, gene expression was not significantly altered after free fatty acid induction. The induction also did not significantly affect *KRT19* expression, an indicator of the cholangiocyte lineage. It is noteworthy that *SREBF1*, a hepatic marker responsible for lipid homeostasis, was upregulated upon free fatty acid induction. *APOB* expression was elevated, which can be reasoned as the increasing need for the cell to transport excessive lipids (Fig. S5a). This trend was also described in previous cohort studies^49,50^. OA induction did not affect the expression of *PNPLA3*, a well-established gene with identified SNPs that lead to increased MASLD susceptibility (Fig. S5b). In comparison, both HLC and sHLC exhibited higher *PNPLA3* expression than HepG2, which is a common immortalized hepatic cell line with an I148M mutation in PNPLA3^51^. For hallmark hepatic functions, less albumin was secreted by the sHLC compared to HLC (Fig. 3g). This was also abrogated by the gene expression level of *ALB*, where the downregulation was observed in sHLC compared to HLC (Fig. S5a). Low urea production was detected for healthy and steatotic HLCs, with no significant difference among these conditions (Fig. S3b). Triglyceride (TG) is the main form of lipids in the human body, and its biosynthesis largely takes place in the liver. TG and apolipoproteins are assembled in the hepatocytes to form lipoproteins, which are later released into the bloodstream to transport lipids. We detected TG secretion from both HLC and the sHLC at a similar level (Fig. 3h). Taken together, our MASLD model showed a strong steatotic phenotype along with partial functional loss compared to healthy HLCs.

**Fig. 4:**
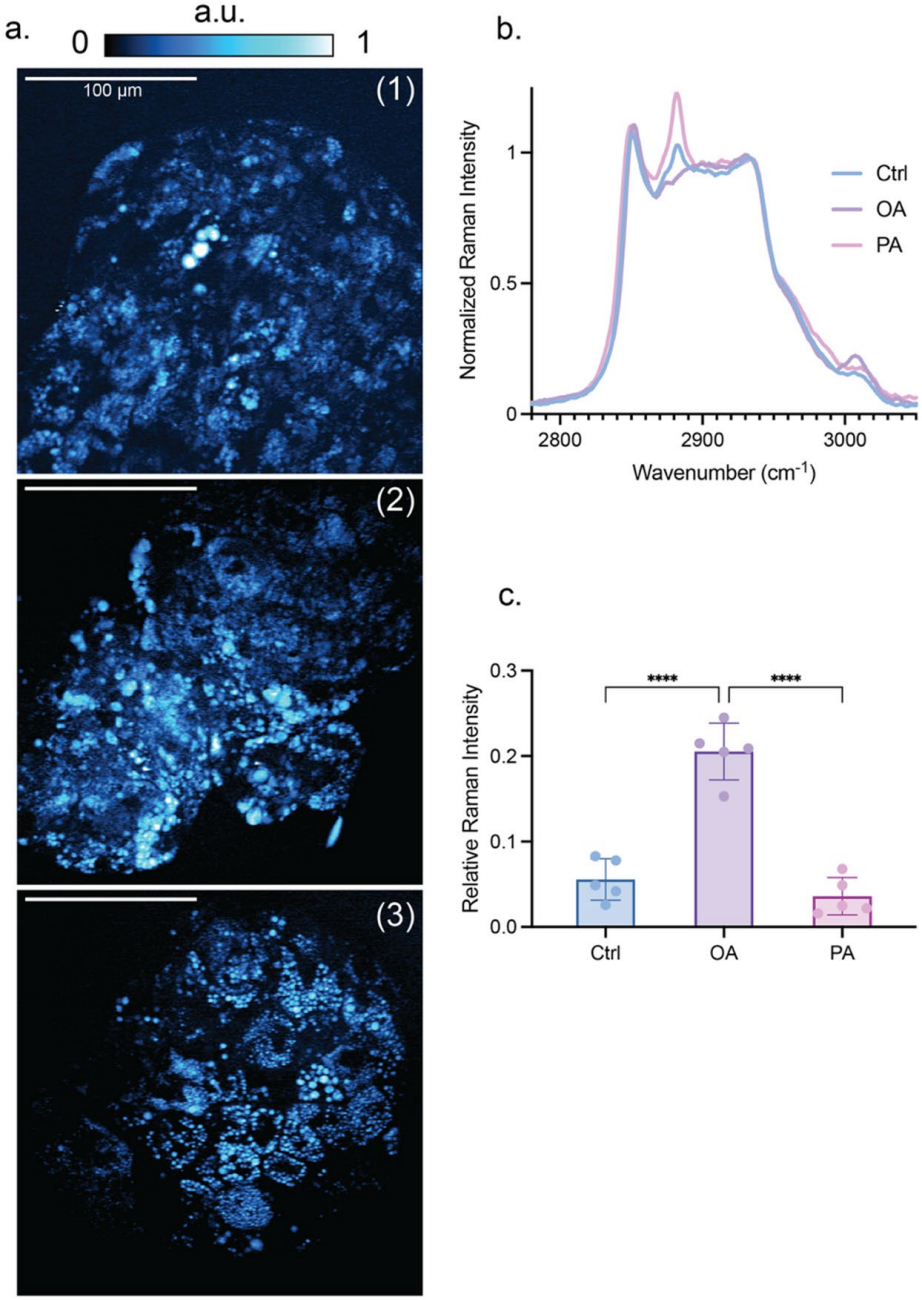
Raman spectroscopy and imaging enable label-free identification of lipid uptake in HLCs. (**a**) SRS images of HA at the 2848 cm^− 1^ symmetric carbon-hydrogen stretching modes of lipids. (1) Control, (2) OA-treated, and (3) PA-treated. Representative results of 3 biological replicates of differentiation, where a.u. is for the arbitrary units. (**b**) Raman spectra of the C-H stretch region of HA from control, OA-treated, and PA-treated. Representative results of 3 biological replicates of differentiation, where *n* = 3 measured HA per condition. (**c**) Raman signal strength of the 3015 cm^− 1^ vibration of unsaturated lipids. Representative results of three biological replicates of differentiation, and *n* = 5 measured HA per condition. The *p*-values of the Shapiro-Wilk test are 0.5312, 0.4931, and 0.3253 for Ctrl, OA, and PA, respectively. The *p*-value is less than 0.0001 for Ctrl vs. OA and OA vs. PA, and 0.5024 for Ctrl vs. PA.

Next, we conducted label-free Raman imaging and micro-spectroscopy measurements to independently validate the cellular uptake and storage of dietary PA and OA. Stimulated Raman scattering (SRS) images of HA treated with PA and OA were recorded at the symmetric methylene stretching mode (2848 cm^− 1^), confirming the presence of intracellular LDs within the HA (Fig. 4a). Averaged Raman spectra of these intracellular LDs displayed a distinct Raman peak at 2890 cm^− 1^ for HA treated with saturated PA, and a characteristic Raman line at 3015 cm^− 1^ for HA treated with unsaturated OA (Fig. 4b). These findings demonstrate that dietary PA and OA were incorporated into the LDs of the respective HA. For instance, the relative intensity of the 3015 cm^− 1^ spectral signature underlines that LDs in OA-induced HA contain a high proportion of unsaturated oleate-based neutral lipids (Fig. 4c).

## Fibrotic stimulation with biochemical cues from the MASLD model

**Fig. 5:**
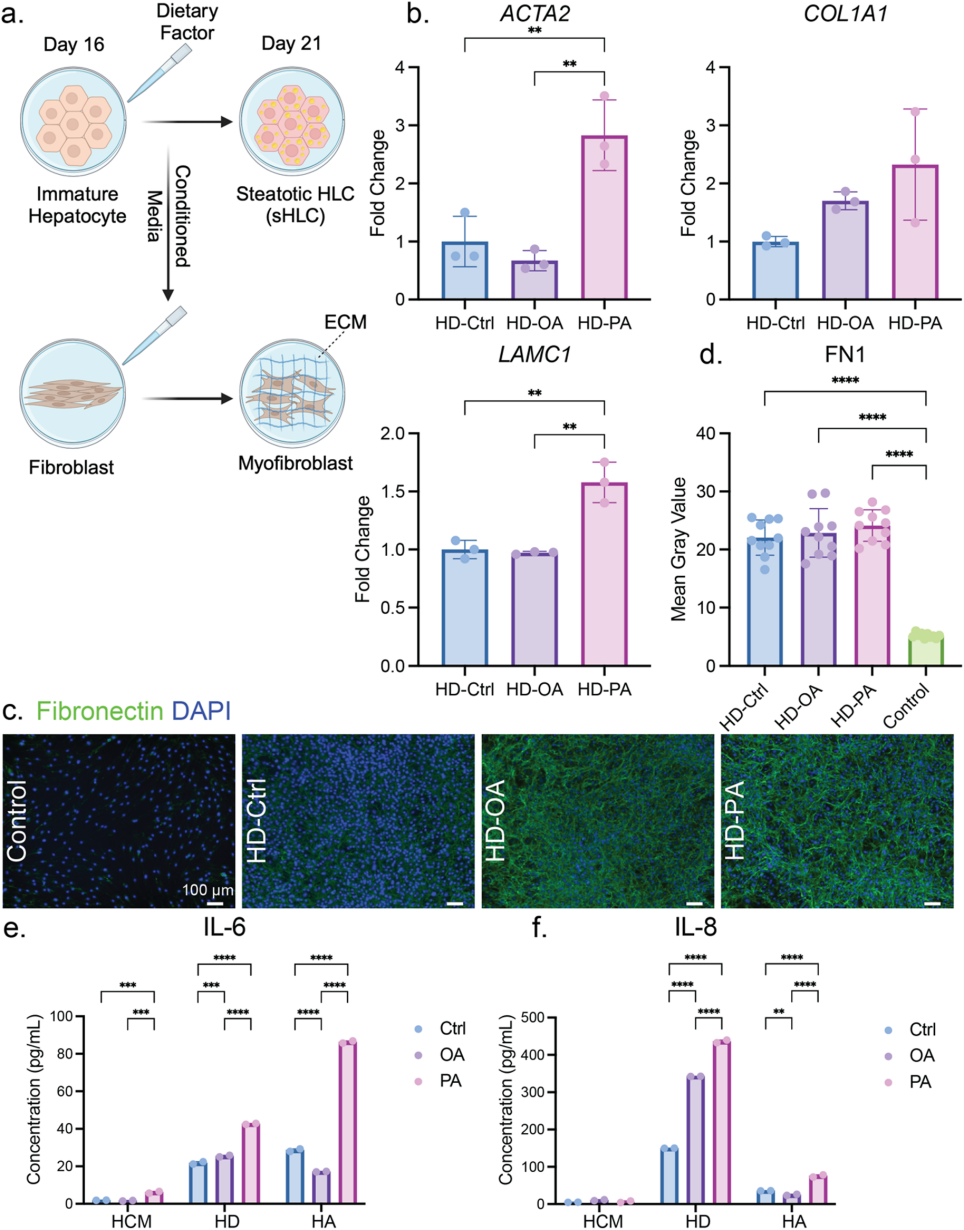
Conditioned media from the MASLD model induce myofibroblastic responses in human dermal fibroblasts. (**a**) Schematics depicting the application of conditioned media from the MASLD model to HDF. (**b**) qRT-PCR results of select myofibroblast markers comparing the HDF activation induced by healthy HLC or the MASLD model. HD-Ctrl, HD-OA, and HD-PA represent the HDF receiving conditioned media from 2D monolayer healthy HLC, OA-induced sHLC, and PA-induced sHLC, respectively. Representative results of 3 biological replicates of differentiation, and each data point entails one technical replicate of the reaction. The *p*-values for HD-Ctrl vs. HD-OA, HD-Ctrl vs. HD-PA, and HD-OA vs. HD-PA are as follows: 0.6549, 0.0056, 0.0024 for *ACTA2*; 0.3446, 0.0627, 0.417 for *COL1A1*; 0.9492, 0.0017, 0.0013 for *LAMC1*. (**c**) Immunofluorescence images of HDF activated by MASLD model (HD-OA and HD-PA) or control (HCM) and their quantification (**d**). Representative results of 3 biological replicates of differentiation, where *n* = 12 images per condition. The *p*-value is < 0.0001 for Control compared with other groups, 0.9254 for HD-Ctrl vs. HD-OA, 0.3988 for HD-Ctrl vs. HD-PA, and 0.7685 for HD-OA vs. HD-PA. The *p*-values of the Shapiro-Wilk test are 0.9104, 0.3658, 0.2885, and 0.7334 for Control, HD-Ctrl, HD-OA, and HD-PA, respectively. Cytokine ELISA results comparing the secretion of IL-6 (**e**) or IL-8 (**f**) from conditioned media (HCM), and HDF activated by 2D (HD) or 3D (HA) MASLD models. Representative results of 3 biological replicates of differentiation, and each data point entails one technical replicate of the reaction. For IL-6, the *p*-values for Ctrl vs. OA, Ctrl vs. PA, and OA vs. PA are as follows: 0.9302, 0.0002, 0.0001 for HCM; 0.0005, < 0.0001, < 0.0001 for HD; < 0.0001, < 0.0001, < 0.0001 for HA. For IL-6, the *p*-values for Ctrl vs. OA, Ctrl vs. PA, and OA vs. PA are as follows: 0.1294, 0.8382, 0.2934 for HCM; < 0.0001, < 0.0001, < 0.0001 for HD; 0.0048, < 0.0001, < 0.0001 for HA.

We next leveraged our established MASLD model to investigate the progression to MASH or liver fibrosis. Conditioned media were collected from the cells undergoing maturation towards HLC or sHLC phenotypes in both 2D (denoted as HD-Ctrl, HD-OA, and HD-PA) and 3D (denoted as HA-Ctrl, HA-OA, and HA-PA). To simulate the crosstalk between hepatic and stromal cells in healthy and diseased settings, conditioned media were applied to human dermal fibroblasts (HDF), a proxy for liver-resident stellate cells, which rapidly lose their phenotype in vitro upon propagation, due to their mechanical sensitivity for myofibroblast activation (Fig. 5a)^52,53^. One metric of myofibroblast induction entails increased deposition of ECM proteins, including collagen I and fibronectin (FN1). After HDF stimulation with conditioned media from our MASLD model, we found upregulation in the gene expression of *Col1A1*, which encodes a subunit of collagen I (Fig. 5b). This was also corroborated by staining for Collagen I, in which the crucial ECM component was found deposited more in HDFs activated by MASLD models than in HDFs treated with transforming growth factor β (TGFβ), a growth factor commonly used to induce fibrosis (Fig. S6). Meanwhile, fibronectin deposition increased in HDFs stimulated by conditioned media from 2D sHLC compared to the control, where no conditioned media was used but HCM alone (Fig. 5c, d). Similarly, we found bolstered fibronectin deposition in HDF stimulated by our 3D MASLD model compared to the negative control of HCM, yet less abundant in contrast to the positive control of TGFβ stimulation (Fig. S7). To rule out the effect of excessive free fatty acids in the conditioned media, we used HCM supplemented with either OA or PA to culture HDF, at the same dosage and duration as those used to induce the MASLD model in HLCs. We confirmed that the excessive free fatty acid did not increase FN1 deposition, if not further diminishing it for the case of OA (Fig. S8). For other myofibroblast markers, the expression of Actin alpha 2, smooth muscle (*ACTA2*, encoding αSMA) was increased in response to conditioned media. Interestingly, we found PA-specific enrichment of laminin subunit gamma 1 (*LAMC1*), which can interact with integrin-alpha V (ITGAV), a key regulator of liver fibrosis that mediates its progression (Fig. 5b)^54,55^.

Inflammation is always concomitant with MASH and the later stages of liver diseases. To this end, we screened for cytokines to investigate whether our MASLD model could trigger inflammation. For both Interleukin-6 (IL-6) and IL-8, almost no secretion was detected for HLC or sHLC. However, when the conditioned media were applied to HDF, both IL-6 and IL-8 revealed a significant increase when the HDF was stimulated by the PA-induced MASLD model (Fig. 5e, f). Together, these results show that biochemical cues derived from our iPSC-derived MASLD model can elicit inflammation and fibrosis in HDF, further propelling our system as a robust model to study various stages of steatotic liver disease.

**Fig. 6:**
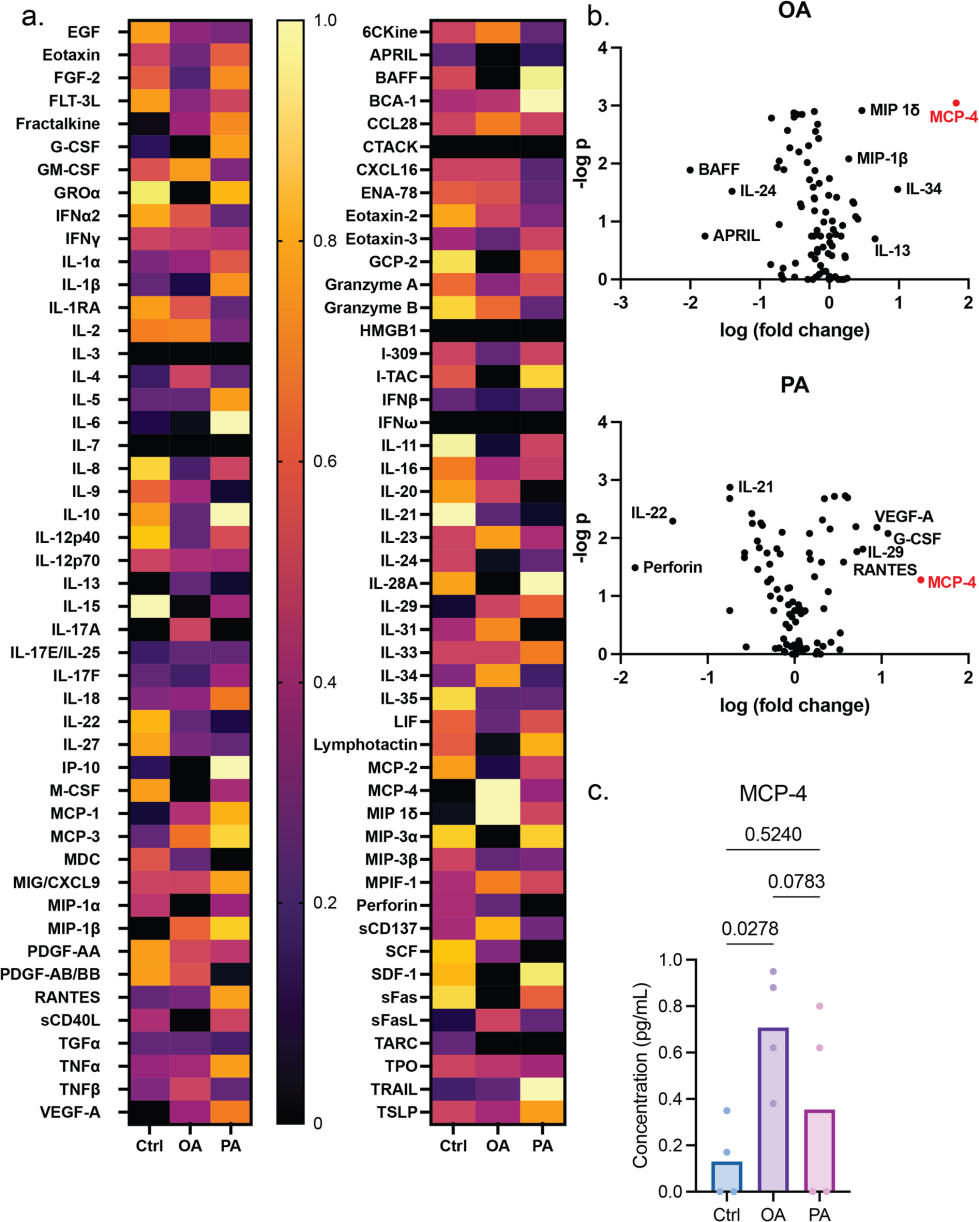
Exploring hepatokines in conditioned media. Multiplex assay for cytokine secretion from 2D monolayer HLC or MASLD models. (**a**) Heatmap and (**b**) volcano plot showing differential cytokine expression among media collected from 2D HLC or MASLD models at Day 21. Collective results of 4 biological replicates of differentiation. (**c**) The concentration of MCP-4 in healthy HLC compared to that upon OA or PA induction. Results of 4 biological replicates of differentiation, and each data point entails 2 technical replicates of the reaction. All *p*-values are labeled above for every comparison between two conditions.

We further investigated which cytokines are responsible for HDF activation. A variety of 96 cytokines was screened for planar HLC, OA-induced sHLC, and PA-induced sHLC. Conditioned media were collected from 4 biological replicates for the multiplex assay, and based on the analysis, we identified several cytokines that exhibited differential secretion levels in sHLCs compared with HLCs (Fig. 6). Among these cytokines, we were able to identify C–C motif chemokine ligand 13 (CCL13, also known as MCP-4)^56,57^ as the major hepatokine with the most drastic upregulation in both OA and PA induced MASLD model (Fig. 6c). MCP-4 is known to be associated with fibrosis in general, yet we have not found any reports claiming its involvement in MASLD progression to date.

Additionally, we discerned the top 5 cytokines with increase in relative secretion from either the OA or the PA induced 2D MASLD model (Fig. S9), and they have all been reported to be correlated with the development or progression of MASLD, namely C–C motif chemokine ligand 4 (CCL4, also known as Macrophage inflammatory protein-1 beta (MIP-1β))^59^, C–C motif chemokine ligand 5 (CCL5, also known as RANTES)^30,58^, C–C motif chemokine ligand 15 (CCL15, also known as Macrophage inflammatory protein-1 delta (MIP-1δ))^59^, Colony stimulating factor 3 (CSF3, also known as Granulocyte-colony stimulating factor (G-CSF))^60^, IL-13^61^, IL-29^62^, IL-34^63^, and Vascular endothelial growth factor A (VEGF-A)^64,65^. Therefore, our modeling system can be a tool to investigate the paracrine effects that contribute to the progression of MASLD.

## Hepatokine induces fibrotic responses in HDF

**Fig. 7:**
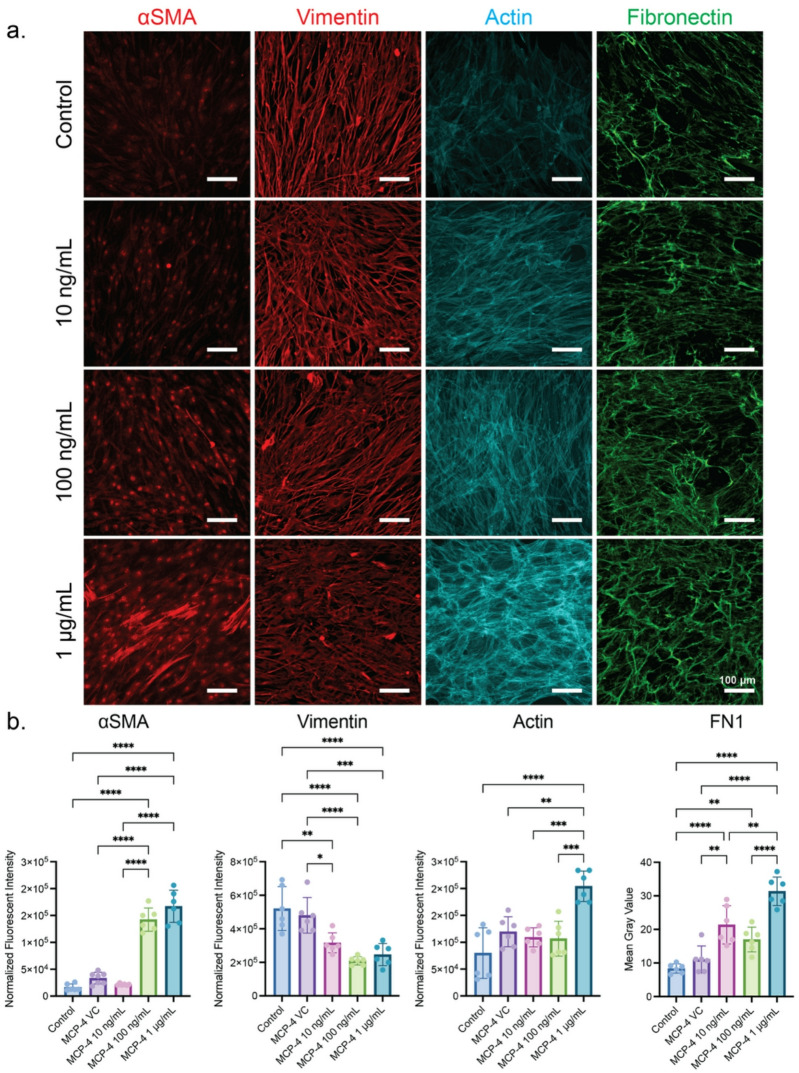
MCP-4 induces fibrotic responses in HDF. (**a**) Representative IF images from 3 biological replicates of different HDF passages treated with varying concentrations of MCP-4. (**b**) The quantification of IF images for αSMA, vimentin, and actin was achieved by normalizing the total IF intensity to the number of cells, and the quantification for fibronectin was achieved by measuring the fluorescent intensity (mean gray value) per image. Each data point represents one image, with a minimum of 3 technical replicates per biological replicate.

To further investigate the effects of the identified hepatokines in our 2D MASLD model, we treated HDF with varying concentrations of MCP-4 and RANTES. As the concentration of MCP-4 increased, a higher IF intensity was observed for the myofibroblast marker αSMA in HDF (Fig. 7). Furthermore, with 1 µg/mL of MCP-4, the HDFs began to exhibit the incorporation of αSMA into the actin fibers and the formation of stress fibers (Fig. 7a). Together, these suggest that MCP-4 was activating the HDFs to a myofibroblast state with the ability to impart increased contractile forces^66^. Additionally, HDFs showed decreased vimentin expression as MCP-4 concentration increased (Fig. 7). This decrease in vimentin, coupled with the stiff culture environment of the polystyrene well plate, indicates that the HDFs could be imparting increased contractile forces^67^. In further support of our MASLD model, HDFs treated with increasing concentrations of MCP-4 showed an increase in fibronectin production (Fig. 7). Altogether, these results indicate that MCP-4 primed the HDFs, increasing their contractility. In the context of liver fibrosis, this increased contractability would allow the HDF to further remodel their surrounding ECM and cause enhanced stiffening of their encapsulating tissue, which could then act as a positive feedback loop for HDF activation^66^. In contrast to MCP-4 treatment, HDFs treated with increasing concentrations of RANTES alone triggered no significant change in vimentin, actin, or fibronectin levels (Fig. S10). Only a higher αSMA intensity was recorded in HDF treated with 100 ng/mL RANTES (Fig. S10). These findings suggest that when the HDF was treated individually with the identified hepatokines, MCP-4 appeared to be more effective at generating fibrotic responses than the other top-hit hepatokines.

## Discussion

Various in vitro techniques utilizing HLCs derived from hiPSCs have been adopted to model MASLD, one of the most common liver disorders. Through the administration of dietary factors, including free fatty acids abundant in MASLD patients^32,33^, in vitro models have accelerated insight into how nutrients^35^, nonparenchymal cell types^34^, and metabolic pathways^36^ contribute to hepatic steatosis and fibrotic progression. Furthermore, the implementation of patient-derived^37^ and CRISPR-edited hiPSCs, along with tunable biomaterials, has further unveiled the role of ancestry, allele-specific SNPs, and extracellular mechanics^38,39^ in the pathogenesis of MASLD and fibrotic processes^40,41^. While these approaches are promising in providing a comprehensive model that captures the complexity of heterotypic cellular interactions and ECM composition in liver disorders, there remains limited understanding of how hepatic lipid injury contributes to fibrotic processes. Here, we demonstrate the ability to generate functional HLCs in 2D and 3D differentiation conditions, which can induce a fibrotic phenotype in stromal cells upon stimulation with dietary factors. Importantly, by measuring the secretome of HLCs exposed to OA and PA conditions, we were able to capture a unique signature of hepatokines mirrored in clinical observations of MASH. Collectively, our tractable system demonstrates the utility of hiPSC-derived HLCs for modeling aspects of MASLD in addition to identifying novel factors implicated in liver disease.

Admittedly, there are several limitations to this study, including deriving HLCs that have comparable functional phenotypes to the PHHs. This remains a pervasive obstacle in the field, even after the manipulation of varied differentiation cocktails, culture substrates, and 3D organoid approaches^43^. In addition, our MASLD model is confined to just a subset of cells that comprise the liver lobule, while other cell types, including cholangiocytes, Kupffer cells, hepatic stellate cells (HSCs), and liver sinusoidal endothelial cells, could provide additional heterotypic cell signaling that can improve hepatic functionality, and features that phenocopy natural liver tissue^68^. We considered a wide variety of dietary factors to initiate the induction of our MASLD model, whereas, unlike the actual diet, we did not excavate further how different dietary factors would contribute to MASLD pathogenesis in combination. Nonetheless, we provide a comprehensive analysis of how hepatic functions are attenuated upon induction of the MASLD. Moreover, our approach provides a more controlled microenvironment, enabling the exploration of the crosstalk from hepatic to non-parenchymal cells. Although we incorporated HDFs as a substitute for HSCs in this study, we observed an inflammatory and fibrotic phenotype activated by paracrine cues elicited from our MASLD model. While established hiPSC-derived MASH or fibrosis models have emphasized direct signaling among cells generated by human liver organoids consisting of multiple liver cell lineages, the ratio of non-parenchymal cell composition is varied and challenging to control^34,39,69^. Furthermore, while HSCs reside in the space of Disse and have some degree of cytoplasmic processes that directly contact hepatocytes, they comprise only 5–8% of all liver cells^70^. Given that hepatocytes are about 10-fold more abundant than the HSCs in the liver, a majority of heterotypic interactions between hepatic and HSCs occur through paracrine effects. Alternative to utilizing the Transwell to allow feedback in intercellular crosstalk^41^, we administered the CM to focus on the unilateral signaling from the hepatocytes to the stromal population. To this end, we identified a range of chemokines and cytokines induced by dietary factors, providing a platform to disentangle the roles of individual hepatokines in fibrotic processes. It is noteworthy that the multiplex assay was performed only on our 2D MASLD model, which has been used in all experiments reported here for fibrotic stimulation, and we have yet to identify any similarities or differences in hepatokine secretion compared to the 3D MASLD model.

The saturated free fatty acid PA was shown to reduce the HLC viability (Fig. 3b), which can be a contributing factor to MASLD progression^71^. In the PA-induced CM, we also found apoptotic or necrotic cytokines (TNF-α, IL-1α, IL-1β, IL-18, IL-33) that may play a role in the paracrine signaling towards MASLD progression (Fig. 6a). Further validation of administering these cytokines individually or combinatorially will explain the extent hepatocyte death is associated with MASLD progression. We were able to monitor the TG secreted by the HLC rather than the total quantity produced, yet no significant difference was observed upon induction. Since we already observed the formation of LDs in sHLC by brightfield imaging and BODIPY staining, it can be inferred that excessive TG was trapped in the sHLC, leading to steatosis and associated functional reductions. Moreover, the upregulation of *APOB* expression indicated a tendency to export redundant TG in sHLC, however our MASLD model didn’t reflect this process. It is noteworthy that we repetitively observed cells with large vacuoles consistent with lipid accumulation in the HA induced by OA (Fig. 3d). This may be the proximity to a diagnostic feature of MASH termed hepatocellular ballooning, yet further validation is needed to corroborate this hypothesis^47^.

With improved engineering tools and advanced modeling systems, including microfluidics^72–74^, 3D bioprinting^75^, and organoid technology, we envision the construction of in vitro liver tissues with physiologically accurate zonation, capturing the complexity of native tissue that can be derived solely from hiPSCs in the future. In addition to these advances, culture systems focused on mimicking the extracellular niche will facilitate new advances in models of liver development and disease. For example, fine-tuning the physical properties of the ECM (i.e., stiffness, porosity, etc.) with engineering strategies can improve MASLD models with more authentic mechanosensory cues. Finally, with the advances in gene editing, we can tailor SNPs that were shown to be correlated with MASLD development, such as the well-known I148M mutation in the PNPLA3 variant, to model the ancestral-specific pathogenesis.

The evaluation of lipid trafficking in HLC is crucial for understanding the pathophysiology of MASLD and related disorders. Label-free Raman imaging, specifically SRS and microspectroscopy, offers a powerful modality for studying the cellular uptake and storage of dietary fatty acids, such as PA and OA. Raman imaging, particularly SRS, enables the label-free visualization of intracellular components by exploiting the vibrational contrast of molecular bonds. In this study, SRS imaging was employed at the symmetric methylene stretching mode (2848 cm^− 1^), which is a prominent marker for lipid-rich structures. The ability to detect these intracellular LDs without the need for external labels using Raman microspectroscopy provides detailed spectral information that can distinguish among different lipid categories within LDs. Averaged Raman spectra of intracellular LDs revealed distinct peaks corresponding to the types of fatty acids incorporated.

The differential incorporation of PA and OA into HLCs provides a novel perception of lipid metabolism and storage mechanisms in hepatocytes. Saturated fatty acids like PA are known to induce lipotoxicity and contribute to the development of steatosis, a hallmark of MASLD. In contrast, unsaturated fatty acids like OA are generally considered less harmful and can even mitigate some of the detrimental effects of saturated fat^76^. By elucidating the specific origin of these fatty acids in HLC, Raman imaging can contribute to a deeper understanding of lipid metabolic processes and their dysregulation in MASLD. Integrating Raman imaging with other omics technologies could also provide a more comprehensive view of the metabolic alterations in MASLD. Collectively, our MASLD model demonstrates the ability to replicate diet-induced liver injury in HLCs. This provides new insight into the specific soluble cues in the liver that impact fibrotic activation, a key factor contributing to long-term liver injury. This system has the potential for practical application in drug screening through high-throughput fabrication of HAs and mass testing of multiple drug candidates.

## Methods and materials

### Cell culture

 Human iPSCs were purchased (Gibco # A18945, Gibco, CA, USA) and cultured feeder-free on the polystyrene well plate (CELLTREAT Scientific Products, MA, USA) coated with 4% Vitronectin XF (STEMCELL Technologies, Vancouver, Canada). Another iPSC line, namely KOLF iPSC, was generously provided by Dr. Edwin Monuki. All experiments in this manuscript were based on the iPSC line purchased from Gibco unless specified otherwise. iPSCs were maintained in mTeSR1 medium (STEMCELL Technologies) at 37 °C with 5% CO_2_ and 95% air. iPSCs were passaged when at least 90% of the cell colonies were compact and almost in contact with each other. After phosphate-buffered saline (PBS, Thermo Fisher Scientific, MA, USA) wash, the iPSC was detached by incubating at 37 °C for 3 to 5 min with a thin film of ReLeSR (STEMCELL Technologies). New mTeSR1 was added to the iPSC, and the detached cells were transferred to a 15 mL conical tube (CELLTREAT Scientific Products). The cells in the tube were centrifuged at 300 g for 5 min, and mTeSR1 was reapplied to the cells after aspirating the supernatant. iPSCs were then seeded into the precoated well plate with 10 µM Y-27,632 (Tocris Bioscience, Bristol, UK). HepG2 cells were obtained from ATCC. Human dermal fibroblasts were purchased from PromoCell (Heidelberg, Germany). Both HepG2 and HDF were cultured as previously described^74^.

### Definitive endoderm differentiation

 The iPSC was differentiated towards the definitive endoderm using the STEMdiff Definitive Endoderm Kit (STEMCELL Technologies) on the well plate where the iPSC had been maintained. When iPSC reached the confluence of around 50%, Medium 1 supplemented with 1% CJ and 1% MR was applied to the cells for 24 h on day 1. From days 2 to 4, cells were cultured in Medium 2 supplemented with 1% CJ, and the medium was changed every day. Both Medium 1 and 2 were prepared according to the protocol from the manufacturer. Definitive endoderm was derived on day 5.

### HLC differentiation

 The definitive endoderm was passaged to a new well plate coated with 4% Vitronectin XF to continue differentiation towards HLC. After PBS wash, the endoderm cell was detached by incubating at 37 °C for 5 min with TrypLE (Thermo Fisher Scientific). New Dulbecco’s Modified Eagle’s Medium/Nutrient Ham’s Mixture F-12 (DMEM/F-12, STEMCELL Technologies) was added to the cells, and the detached cells were transferred to a 15 mL conical tube. The cells in the tube were centrifuged at 300 g for 5 min, and Medium A (DMEM/F-12 as the basal medium, 10% KnockOut Serum Replacement (Thermo Fisher Scientific), 1% DMSO (Sigma-Aldrich, MO, USA), 0.5% GlutaMAX (Gibco), 0.5% MEM Non-Essential Amino Acids (Gibco), and 100 ng/mL HGF (PeproTech, NJ, USA)) was applied to the cell after aspirating the supernatant. The endoderm cell was then seeded to the precoated well plate and maintained at 37 °C with 5% CO_2_ and 95% air. Medium was changed every day with Medium A from day 6 to day 12. From day 13 to day 15 the cells were maintained in Medium B (DMEM/F-12 as basal medium, 10% KnockOut Serum Replacement, 40 ng/mL Dexamethasone (Thermo Fisher Scientific) in 1% DMSO, 0.5% GlutaMAX, 0.5% MEM Non-Essential Amino Acids, and 100 ng/mL HGF) with daily medium change. Cell passage similar to day 5 might occur on either day 13 or day 16, depending on cell confluence or the following procedure. At this optional passage, the cells were detached for prolonged treatment of TrypLE for at least 15 min, and then seeded at the density of about 3 × 10^4^ cells/cm^2^ on the well plate coated with 4% Vitronectin XF or 10% Basement Membrane Extract (BME, R&D Systems, MN, USA). From day 16 to day 20, the cells were maintained in Hepatocyte Culture Medium (HCM, Lonza, Basel, Switzerland), excluding rhEGF and supplemented with 20 ng/mL Oncostatin-M (PeproTech). The medium was changed every day until the derivation of HLC on day 21. The differentiation process is consistent with our previously published work^77^.

### Hepatic aggregate formation

 HA was generated in the Aggrewell 400 Microwell Culture Plate (STEMCELL Technologies) per the manufacturer’s protocol. Differentiating cells at the hepatoblast stage (days 13 to 16) were collected as previously described, and were seeded into the Aggrewell at a density of 200 cells per microwell (24,000 cells per well). HCM was added to a total volume of 2 mL per well. The Aggrewell was treated with 0.5 mL Anti-Adherence Rinsing Solution (STEMCELL Technologies) per well, and centrifuged at 1300 g for 10 min. The Aggrewell was then washed with 2 mL DMEM/F-12 per well prior to seeding. After gently mixing the seeded cells, the Aggrewell was centrifuged at 100 g for 3 min, and then incubated at 37 °C with 5% CO_2_ and 95% air. Medium B was used for days 13 to 15, and HCM with OSM was used for days 16 to 21 as previously described. For the daily medium change, 1 mL of medium was slowly removed from the side of each well, and 1 mL of fresh medium was slowly added from the side of each well. HA was collected by day 21. A serological pipette was used to gently mix the medium in the well to suspend HA, and then all liquid was transferred to a 15 mL conical tube. Each well was washed twice with 1 mL of DMEM/F-12 and then transferred to the corresponding 15 mL conical tube. The tube was set aside for the HA to settle down at the bottom for at least 5 min. The supernatant was aspirated, and the pellet was washed with PBS before further use.

### MASLD model induction

 Dietary factors were used to induce our MASLD model during the maturation stage of HLC differentiation from day 16 to day 21. Glucose (Sigma-Aldrich), insulin (Sigma-Aldrich), oleic acid (Sigma-Aldrich), palmitic acid (Sigma-Aldrich), and cholesterol (Sigma-Aldrich) were supplemented to the HCM with OSM at a range tested in Fig. S4. The medium was changed every day during the induction, and the cells were maintained at 37 °C with 5% CO_2_ and 95% air. For 3D MASLD induction, dietary factors in the fresh medium were prepared twice more concentrated to reach the designated concentrations for the HA after the daily medium change.

### Fibrotic stimulation

 Culture medium supernatant was collected from the 2D or 3D MASLD model every day from day 17 to day 21, and centrifuged at 4 °C to remove any cell debris. The supernatant was transferred to a new 1.5 mL tube and stored at − 20 °C before use. HDF was seeded onto 24-well plates without any treatment at a density of 20,000 cells per well. After 48 h, which is day 1 for fibrotic stimulation, conditioned media from day 17 of hepatic maturation or sHLC derivation were applied to the HDF. For day 2 of fibrotic stimulation, after removing the media in the wells of HDF, conditioned media from day 18 of hepatic maturation or sHLC derivation was applied to the HDF. This lasted every day until day 5 of fibrotic stimulation, when conditioned media from day 21 of hepatic maturation or sHLC derivation were applied to the HDF after the removal of previous media. On day 6 of fibrotic stimulation, culture medium supernatant was collected and stored at − 20 °C for later use, while the cells were washed with PBS for further experiments. During the fibrotic stimulation, cells were maintained at 37 °C with 5% CO_2_ and 95% air. HCM was used as the negative control, and HCM supplemented with 5 ng/mL TGFβ (PeproTech) was used as the positive control. The media were changed daily for both controls.

### Immunofluorescence

For cells cultured on the well plate, 4% paraformaldehyde (PFA, Santa Cruz Biotechnology, TX, USA) was added to each well for 15 min after PBS removal. The cells were then washed with PBS at least twice after fixation, and treated with Blocking Buffer (0.5% Bovine serum albumin (BSA, Sigma-Aldrich), 0.1% Triton X-100 (Sigma-Aldrich) in PBS) for permeabilization and blocking at room temperature for an hour. Primary antibodies were diluted according to their protocol in the Blocking Buffer, and then applied to cells for overnight incubation at 4 °C on a shaker. After incubation with primary antibodies, the cells were then washed at least twice with PBS before staining with secondary antibodies. Secondary antibodies were diluted according to their protocol in the Blocking Buffer, along with DAPI (1:1000, Thermo Fisher Scientific) and phalloidin (1:500, Thermo Fisher Scientific). All antibodies we used were listed in Table S1. Alternatively, BODIPY (4,4-Difluoro − 1,3,5,7,8-Pentamethyl-4-Bora-3a,4a-Diaza-s-Indacene, Thermo Fisher Scientific) was dissolved in ethanol to make a stock solution of 1 mM, and diluted at 1:50 when used with DAPI and phalloidin. The solution was applied to cells for one hour of incubation at room temperature on a shaker. The stained cells were washed at least twice with PBS, and then visualized on the BZ-X810 Inverted Fluorescence Phase Contrast Microscope (Keyence, Osaka, Japan). For aggregates, 4% PFA was added for at least 15 min after PBS removal. The aggregates were then washed with PBS before incubation in the Blocking Buffer for an hour at room temperature. Diluted primary antibodies were applied to the aggregates for overnight incubation at 4 °C. After PBS wash, diluted DAPI and phalloidin with secondary antibodies or BODIPY were applied to aggregates for overnight incubation at 4 °C. The stained aggregates were washed with PBS and then transferred to the glass bottom coverslip (Ibidi, Gräfelfing, Germany) to be visualized on the Fluorescence Microscope or Zeiss LSM 900 Airyscan 2 Confocal Microscopy (Carl Zeiss AG, Oberkochen, Germany). Image processing and quantification were achieved using software FIJI (NIH, MD, USA) and ZEN (Carl Zeiss AG).

### PAS staining

 The Periodic Acid-Schiff Staining System (Sigma-Aldrich) was applied to visualize the glycogen biosynthesis and storage. On day 21, the medium was removed for HLC cultured on the well plate before fixation with 4% PFA for 15 min, and then the cells were washed with water for 1 min. For aggregates, HA was collected and fixed with 4% PFA for at least 15 min, then transferred to the glass-bottom coverslip to be washed for 1 min. The periodic acid solution was added to the fixed sample for 5 min at room temperature, and then washed three times with water. Schiff’s Reagent was added to the sample for 15 min at room temperature, and then washed with water for 5 min. Counterstain was then performed with Hematoxylin solution for 1.5 min, and then washed at least three times with water. The sample was visualized on the Fluorescence Microscope.

### ICG uptake

 The indocyanine working solution was prepared at the concentration of 5 mg/mL by dissolving ICG powder (Sigma-Aldrich) into water. After filtration with the PVDF syringe filter (0.22 μm pore size, CELLTREAT Scientific Products), the working solution was diluted to 1 mg/mL with HCM. The cells to be tested were washed with PBS once, and incubated at 37 °C with 5% CO_2_ and 95% air for 15 min in HCM with 1 mg/mL ICG. Cells were washed three times with PBS before imaging under the Fluorescence Microscope.

### Enzyme linked immunosorbent assay (ELISA)

 To measure the albumin secretion level, culture medium supernatant was collected from the cells at different time points or under different culture conditions and stored at − 20 °C. Coating antibody (Thermo Fisher Scientific) diluted with coating buffer (1.75 g NaHCO_3_ (Sigma-Aldrich), 0.2 g Na_2_CO_3_ (Thermo Fisher Scientific) in 0.5 L water, pH 9.6) at 1:100 was added to the ELISA plate (Stellar Scientific, MD, USA), and incubated at room temperature for 1 h. After washing three times with washing buffer (6.04 g Tris-HCl (Thermo Fisher Scientific), 8.12 g NaCl (VWR, PA, USA), 0.5 mL Tween 20 (Sigma-Aldrich) in 1 L water, pH 8.0), the plate was blocked with blocking buffer (6.04 g Tris-HCl, 8.12 g NaCl, 10 g BSA in 1 L water, pH 8.0) for 1 h at room temperature or overnight at 4 °C. After washing three times with washing buffer, the standards (Sigma-Aldrich) and samples were added to the plate and incubated for 1 h at room temperature. After washing three times with washing buffer, the HRP-conjugated antibody (Thermo Fisher Scientific) diluted with sample diluent (6.04 g Tris-HCl, 8.12 g NaCl, 0.5 mL Tween 20, 10 g BSA in 1 L water, pH 9.6) at 1:100,000 was added to the plate, and incubated in dark for 45 min at room temperature. After washing three times with washing buffer, TMB substrate (Thermo Fisher Scientific) was added to the plate and quenched with 0.5 N HCl (VWR). The absorbance was recorded at 450 nm on the Spark Multimode Microplate Reader (Tecan, Männedorf, Switzerland). The concentrations were calculated based on the standard curve within the same run, and the comparison was made with the assumption that the initial cell seeding density, as well as the cell culture conditions, were the same across all samples. For the comparison between HLC and HA, the concentration was normalized by the initial cell seeding density. For cytokine secretion, IL-6 and IL-8 ELISA kits (Thermo Fisher Scientific) were used for the culture medium supernatant collected on day 6 of fibrotic stimulation per the manufacturer’s protocol.

### CYP3A4 activity assay

 For cells to be tested for CYP3A4 activity, drugs were supplemented to the HCM and administered to the cells twice for a duration of 48 h via daily media change. The dexamethasone (50 µM) was used to induce CYP3A4 activity. CYP3A4 activity was determined with the P450-Glo™ CYP3A4 Assay (Promega, WI, USA) and normalized by cell viability, which was determined with the CellTiter-Glo^®^ Luminescent Cell Viability Assay (Promega). Both experiments were carried out according to the manufacturer’s protocol.

### Urea colorimetric assay

 Culture medium supernatant was collected from the cells and stored at − 20 °C. The secreted urea concentration was determined with Stanbio™ BUN Liquid Reagent for Diagnostic Set (EKF Diagnostics, TX, USA) per the manufacturer’s protocol. The concentrations were calculated based on the standard curve within the same run, and the comparison was made with the assumption that the initial cell seeding density, as well as the cell culture conditions, were the same across all samples. For the comparison between HLC and HA, the concentration was normalized by the initial cell seeding density.

### Triglyceride assay

 Culture medium supernatant was collected from the cells and stored at − 20 °C. The secreted triglyceride concentration was determined with Triglyceride-Glo™ Assay (Promega) per the manufacturer’s protocol. The comparison was made with the assumption that the initial cell seeding density, as well as the cell culture conditions, were the same across all samples.

### Raman spectroscopy

 Fixed HAs were suspended in PBS and placed between two borosilicate coverslips for the Raman microspectroscopy measurements. All samples were blinded before the measurements. Raman spectra were collected on a Renishaw InVia Raman microscope using a 532 nm laser excitation source, a dry 0.75 NA objective lens, and a 1 s signal integration time. The signal was averaged 120 times to improve the signal-to-noise ratio. Raman point measurements in confocal detection mode were carried out at selected locations by focusing the excitation light onto intracellular lipid droplets.

### Stimulated Raman scattering microscopy

 The stimulated Raman scattering (SRS) microscope consisted of a picosecond optical parametric oscillator (picoEmerald OPO, A.P.E. Berlin) synchronously pumped by an 80 MHz, 1031 nm picosecond laser. The signal from the OPO was tuned in the 784.5 to 800 nm range and served as the pump beam in the SRS process, whereas the residual of the 1031 nm beam was used as the Stokes beam. The Stokes beam was intensity modulated at 20 MHz. The pump and Stokes beams were temporally and spatially overlapped on a dichroic mirror and directed to a laser scanner (Fluoview 300 and IX71, Olympus)^78^. The beams were focused onto the sample by a 1.2 NA water immersion objective lens (Olympus). The signal was collected by a 1.4 NA oil immersion condenser (Olympus) in the transmission direction. After passing through a 960 nm shortwave pass filter, the pump beam was detected by a photodiode (S3634, Hamamatsu). The stimulated Raman loss (SRL) signal was extracted by demodulation of the signal with a lock-in amplifier (HF2LI, Zurich Instruments) at 20 MHz. The samples were prepared similarly as in the Raman microspectroscopy experiments, and all samples were blinded before imaging.

### Multiplex assay

 Cell culture medium supernatant was collected from HLC or sHLCs and stored at − 20 °C. The measurement of 96 cytokines was carried out by EVE Technologies (Calgary, Canada). A total of 4 biological replicates were analyzed, with two measurements of technical replicates performed for each sample, and all samples were blinded before measurements.

### Statistical analysis

 All statistical analyses were carried out using the GraphPad Prism version 9 or above (GraphPad Software, CA, USA). Two-tailed *t*-test was applied to compare the means of two groups. One-way ANOVA was used for three or more groups based on one factor, and two-way ANOVA was used to compare the means of groups based on two factors. Turkey’s multiple comparison test was performed for all one-way and two-way ANOVA to determine statistical significance. For statistical significance, the *p*-value of less than 0.05 was considered, where **** for *p* ≤ 0.0001, *** for *p* ≤ 0.001, ** for *p* ≤ 0.01, and * for *p* ≤ 0.05. The Shapiro-Wilk test was performed to assess the normal distribution. If the dataset failed the test for normal distribution, the Kruskal-Wallis test was performed instead of one-way ANOVA, which was the case for Fig. 3e and S4 (except for PA). All error bars in the graphs are the standard deviation (SD). Heatmaps and volcano plots were generated with GraphPad Prism. All experiments have been conducted in at least three biological replicates, where each biological replicate comes from an independent differentiation of a distinct hiPSC passage. The exact number of biological and technical replicates is indicated in the corresponding figure, along with the individual n values representing the sample sizes for each condition where applicable.

### Miscellaneous

 All schematics were created with BioRender.com.

## Supplementary Information

Below is the link to the electronic supplementary material.

Supplementary Material 1

## Data Availability

All data generated in this study to demonstrate the key findings are provided in the manuscript and the Supplementary Information. Any other data supporting this study are available from the corresponding author upon reasonable request.
