# Supplementary material for: Modeling fatty liver disease and progression with stem cell derived hepatocytes
Source: Sci Rep. 2026 Jan 7;16:4835. doi: 10.1038/s41598-025-34762-1 (PMC12873437; doi:10.1038/s41598-025-34762-1)
Supplement: Supplementary file 1 — Supplementary Material 1 [file 41598_2025_34762_MOESM1_ESM.pdf]

Supplementary Information

Modeling Fatty Liver Disease and Progression with Stem Cell Derived Hepatocytes

Yao Wang [1,2], David Berlin [1,2], Yong Li [3], Lok Man Ko [1], Zhenzhu Qi [1], Jiayi Feng [4], Christopher T. Clark [1,2], Diandian Cheng [1,2], Melisa Andrade [1,2], Eric Potma [3], Quinton Smith [1,2,4,5 *]

[1.] Department of Chemical and Biomolecular Engineering, University of California, Irvine, Irvine, CA 92697, USA.
[2.] Sue and Bill Gross Stem Cell Research Center, University of California, Irvine, Irvine, CA 92697, USA.
[3.] Department of Chemistry, University of California, Irvine, Irvine, CA 92697, USA.
[4.] Department of Biomedical Engineering, University of California, Irvine, Irvine, CA 92697, USA.
[5.] Department of Materials Science and Engineering, University of California, Irvine, Irvine, CA 92697, USA

*Corresponding author: Quinton Smith, Ph.D.
Department of Chemical and Biomolecular Engineering,
Sue and Bill Gross Stem Cell Research Center,
University of California, Irvine, CA, United States
Email: quintons@uci.edu

Table

Table S1. List of antibodies and primers.

Reagent	Source	Identifier
Primers		
GAPDH	Thermo Fisher Scientific	4333764F
TBP	Thermo Fisher Scientific	Hs00427620_m1
RNA45S5	Thermo Fisher Scientific	HS03928985_g1
SOX2	Thermo Fisher Scientific	Hs01053049_s1
FoxA2	Thermo Fisher Scientific	Hs05036278_s1
HNF4α	Thermo Fisher Scientific	Hs00230853_m1
AFP	Thermo Fisher Scientific	Hs00173490_m1
ALB	Thermo Fisher Scientific	Hs00609411_m1
SERPINA1	Thermo Fisher Scientific	Hs01097800_m1
APOB	Thermo Fisher Scientific	Hs00181142_m1
KRT19	Thermo Fisher Scientific	Hs00761767_s1
SREBF1	Thermo Fisher Scientific	Hs01088691_m1
PNPLA3	Thermo Fisher Scientific	Hs00228747_m1
ACTA2	Thermo Fisher Scientific	Hs00426835_g1
COL1A1	Thermo Fisher Scientific	Hs00164004_m1
COL4A1	Thermo Fisher Scientific	Hs00266237_m1
LAMC1	Thermo Fisher Scientific	Hs00267056_m1
MYH11	Thermo Fisher Scientific	Hs00975796_m1
Antibodies		
HNF4α	Cell Signaling Technology	3113
AFP	Santa Cruz Biotechnology	sc-8399
ALB	Santa Cruz Biotechnology	sc-271605
A1AT	Abcam	ab207303
EpCam	Cell Signaling Technology	36746
SOX9	Santa Cruz Biotechnology	sc-166505
FN1	Cell Signaling Technology	26836
COL1A1	Cell Signaling Technology	72026
αSMA	Santa Cruz Biotechnology	sc-32251
Vimentin	Santa Cruz Biotechnology	sc-6260
Human Albumin Polyclonal	Thermo Fisher Scientific	A80-129A
Human Albumin Polyclonal, HRP	Thermo Fisher Scientific	A80-129P

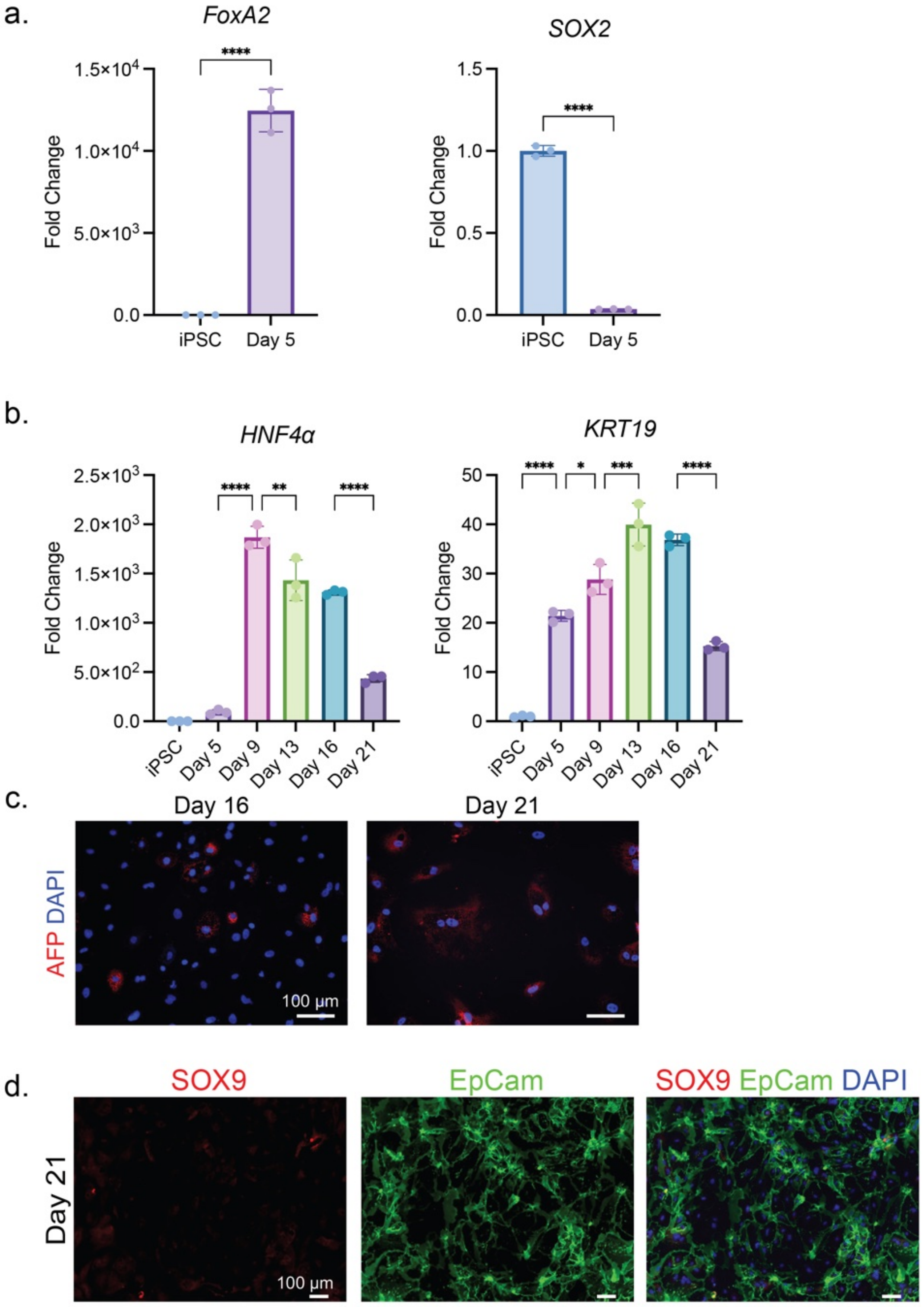

a.
FoxA2
Fold Change
1.5×10^4
1.0×10^4
5.0×10^3
0.0
****
iPSC
Day 5
SOX2
Fold Change
1.5
1.0
0.5
0.0
****
iPSC
Day 5
b.
HNF4α
Fold Change
2.5×10^3
2.0×10^3
1.5×10^3
1.0×10^3
5.0×10^2
0.0
****
**
****
iPSC
Day 5
Day 9
Day 13
Day 16
Day 21
KRT19
Fold Change
50
40
30
20
10
0
****
*
***
****
iPSC
Day 5
Day 9
Day 13
Day 16
Day 21
c.
Day 16
Day 21
AFP DAPI
100 µm
d.
SOX9
EpCam
SOX9 EpCam DAPI
Day 21
100 µm

Figure S1. Characterization of iPSC derived hepatocyte like cells. (a) qRT-PCR results of endoderm marker (*FoxA2*) and pluripotency marker (*SOX2*) before and after definitive endoderm differentiation. Representative results of 3 biological replicates of differentiation, and n = 3 reactions. The p-values are less than 0.0001. **(b)** qRT-PCR results of hepatic development marker (*HNF4α*) and cholangiocyte marker (*KRT19*) at different time points of HLC derivation. Representative results of 3 biological replicates of differentiation, and n = 3 reactions. Unless indicated below, all p-values are less than 0.0001 (****) or larger than 0.9999 (without annotation) for every two adjacent comparisons. For *HNF4α*, p = 0.8445 for iPSC vs. Day 5, p = 0.0016 for Day 9 vs. Day 13, p = 0.6591 for Day 13 vs. Day 16. For *KRT19*, p = 0.0185 for Day 5 vs. Day 9, p = 0.0007 for Day 9 vs. Day 13, p = 0.5851 for Day 13 vs. Day 16. **(c)** Immunofluorescence (IF) images of immature hepatocyte marker (AFP) at days 16 and 21 of HLC differentiation. Representative results of 3 biological replicates of differentiation. **(d)** IF images of hepatic progenitor marker (EpCam) and cholangiocyte marker (SOX9) expressed in HLC. Representative results of 3 biological replicates of differentiation.

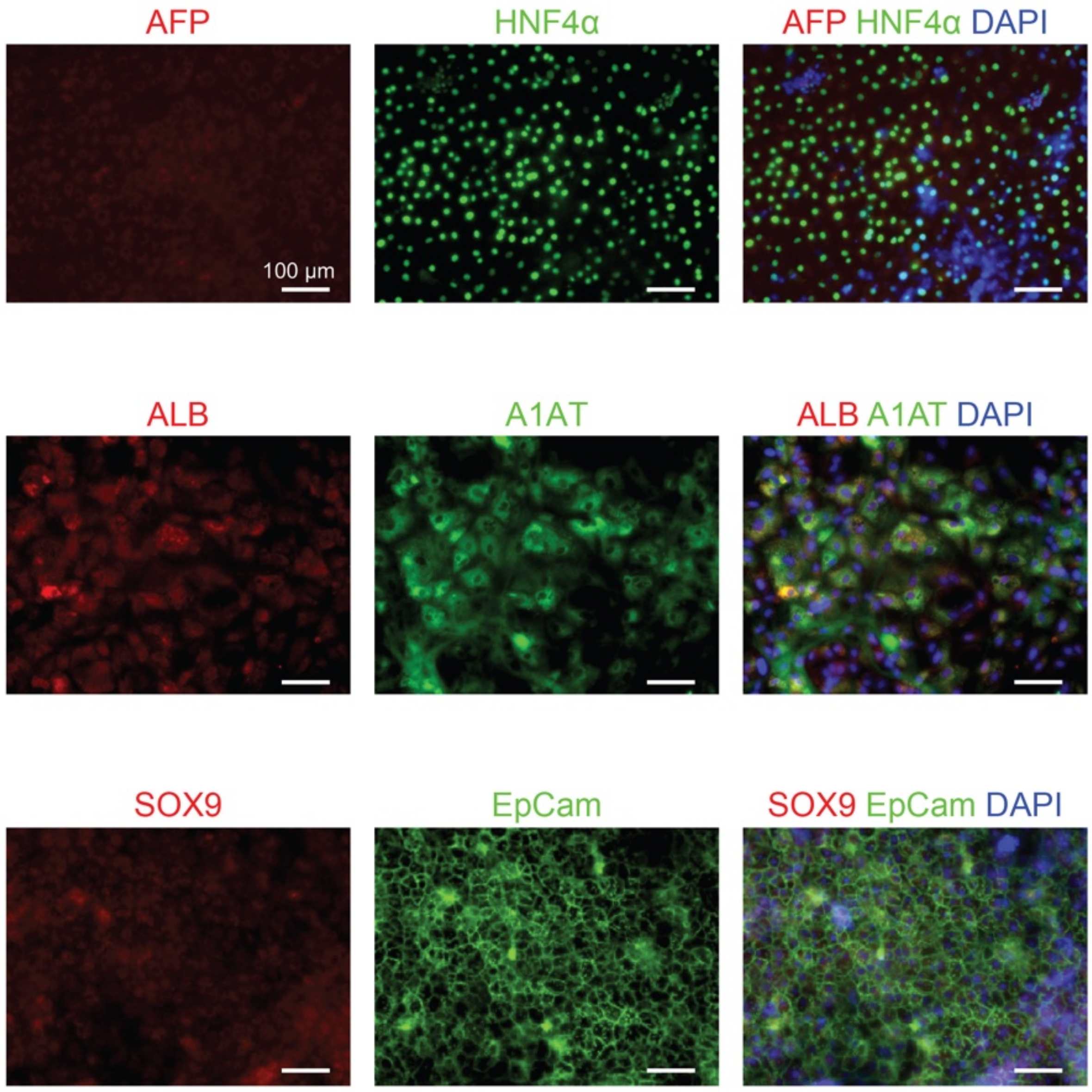

Figure S2. Immunofluorescence characterization. Immunofluorescence images of select haptic markers in HLC differentiated from KOLF iPSC (Day 21). Representative results of 3 biological replicates of differentiation.

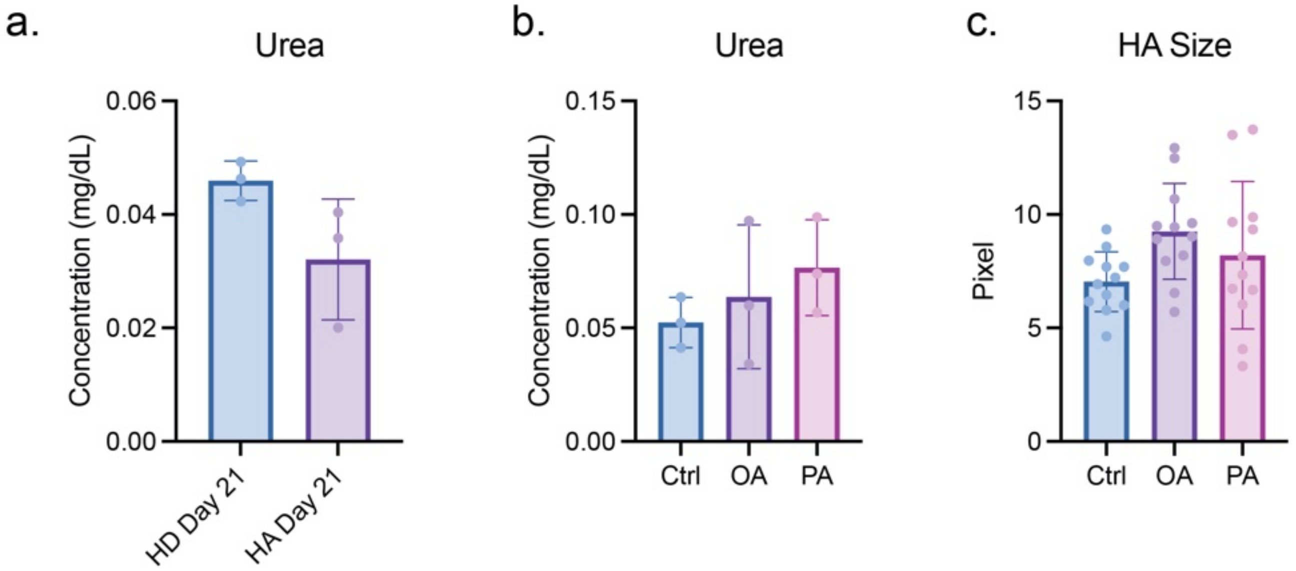

Figure S3. Analysis of 2D and 3D MASLD model. (a) Urea secretion from HLC and HA normalized by initial cell seeding density. Representative results of 3 biological replicates of differentiation, and n = 3 reactions, p = 0.1927. **(b)** Urea secretion from HLC and sHLCs induced by OA or PA. Representative results of 3 biological replicates of differentiation, and each data point entails one technical replicate of the reaction. The p-value is 0.8219 for Ctrl vs. OA, 0.4491 for Ctrl vs. PA, and 0.78 for OA vs. PA. **(c)** The sizes of healthy and induced HAs. Representative results of 3 biological replicates of differentiation, and each data point stands for the size of one HA. The p-values of the Shapiro-Wilk test are 0.9997, 0.7642, and 0.6193 for Ctrl, OA, and PA, respectively. The p-value is 0.0708 for Ctrl vs. OA, 0.4564 for Ctrl vs. PA, and 0.5303 for OA vs. PA.

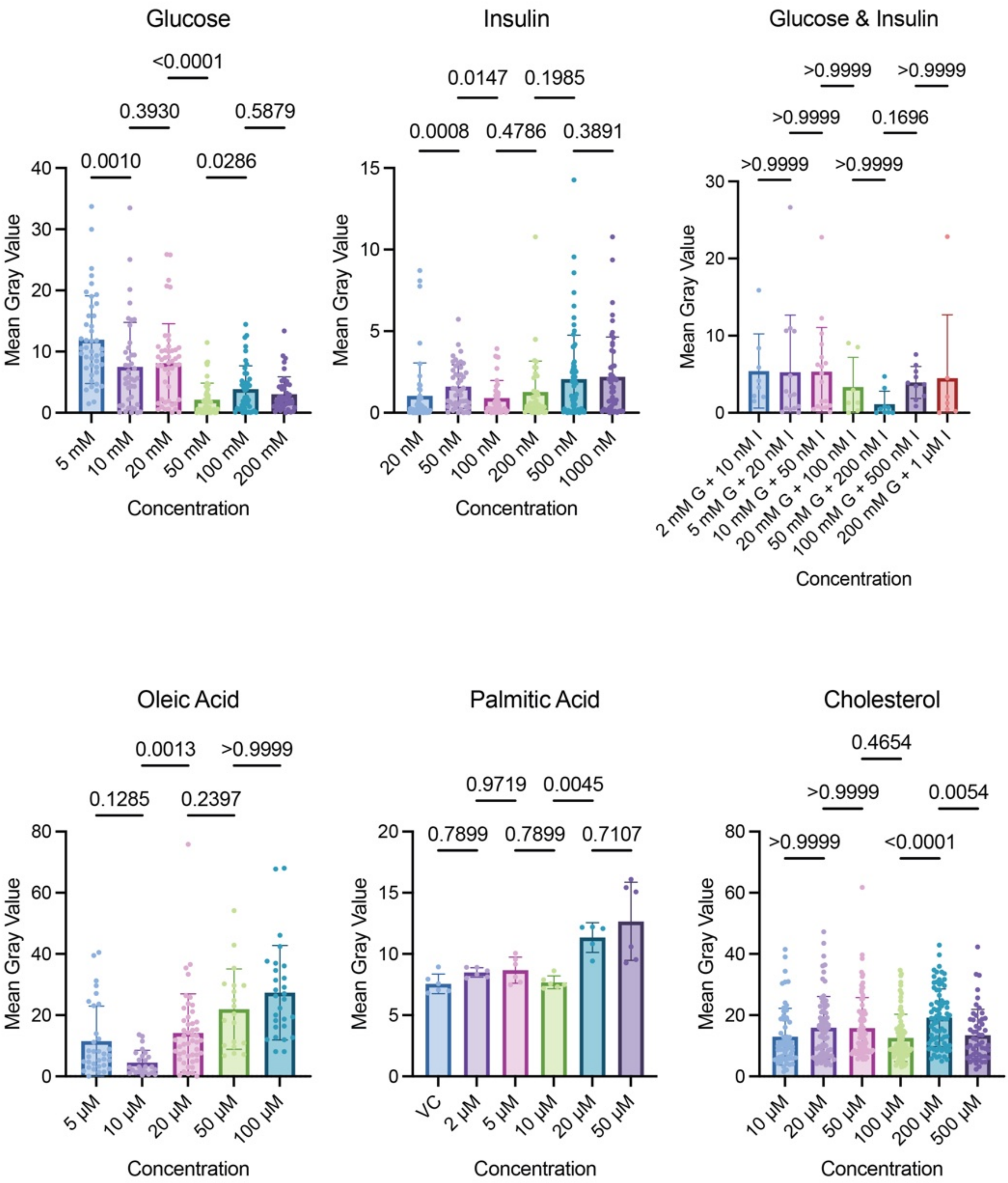
Glucose
<0.0001
0.3930
0.5879
0.0010
0.0286
Mean Gray Value
0
10
20
30
40
5 mM
10 mM
20 mM
50 mM
100 mM
200 mM
Concentration
Insulin
0.0147
0.1985
0.0008
0.4786
0.3891
Mean Gray Value
0
5
10
15
20 nM
50 nM
100 nM
200 nM
500 nM
1000 nM
Concentration
Glucose & Insulin
>0.9999
>0.9999
>0.9999
0.1696
>0.9999
>0.9999
Mean Gray Value
0
10
20
30
2 mM G + 10 nM I
5 mM G + 20 nM I
10 mM G + 50 nM I
20 mM G + 100 nM I
50 mM G + 200 nM I
100 mM G + 500 nM I
200 mM G + 1 μM I
Concentration
Oleic Acid
0.0013
>0.9999
0.1285
0.2397
Mean Gray Value
0
20
40
60
80
5 μM
10 μM
20 μM
50 μM
100 μM
Concentration
Palmitic Acid
0.9719
0.0045
0.7899
0.7899
0.7107
Mean Gray Value
0
5
10
15
20
VC
2 μM
5 μM
10 μM
20 μM
50 μM
Concentration
Cholesterol
0.4654
>0.9999
0.0054
>0.9999
<0.0001
Mean Gray Value
0
20
40
60
80
10 μM
20 μM
50 μM
100 μM
200 μM
500 μM
Concentration

Figure S4. MASLD model optimization. Concentration optimization for the MASLD model using dietary factors independently or jointly, including glucose, insulin, OA, PA, and cholesterol. Representative results of at least 2 biological replicates of differentiation, and each data point entails one technical replicate of the enumerated cell. All p-values are labeled atop for every comparison between two adjacent concentrations. The n values for each condition from lower to higher concentration are as follows: 43, 42, 48, 45, 45, 45 for glucose; 50, 47, 41, 44, 65, 49 for insulin; 8, 14, 18, 7, 9, 10, 7 for glucose and insulin; 33, 25, 48, 19, 28 for OA; 60, 71, 79, 80, 77, 53 for cholesterol; 6, 6, 6, 6, 5, 6 for PA.

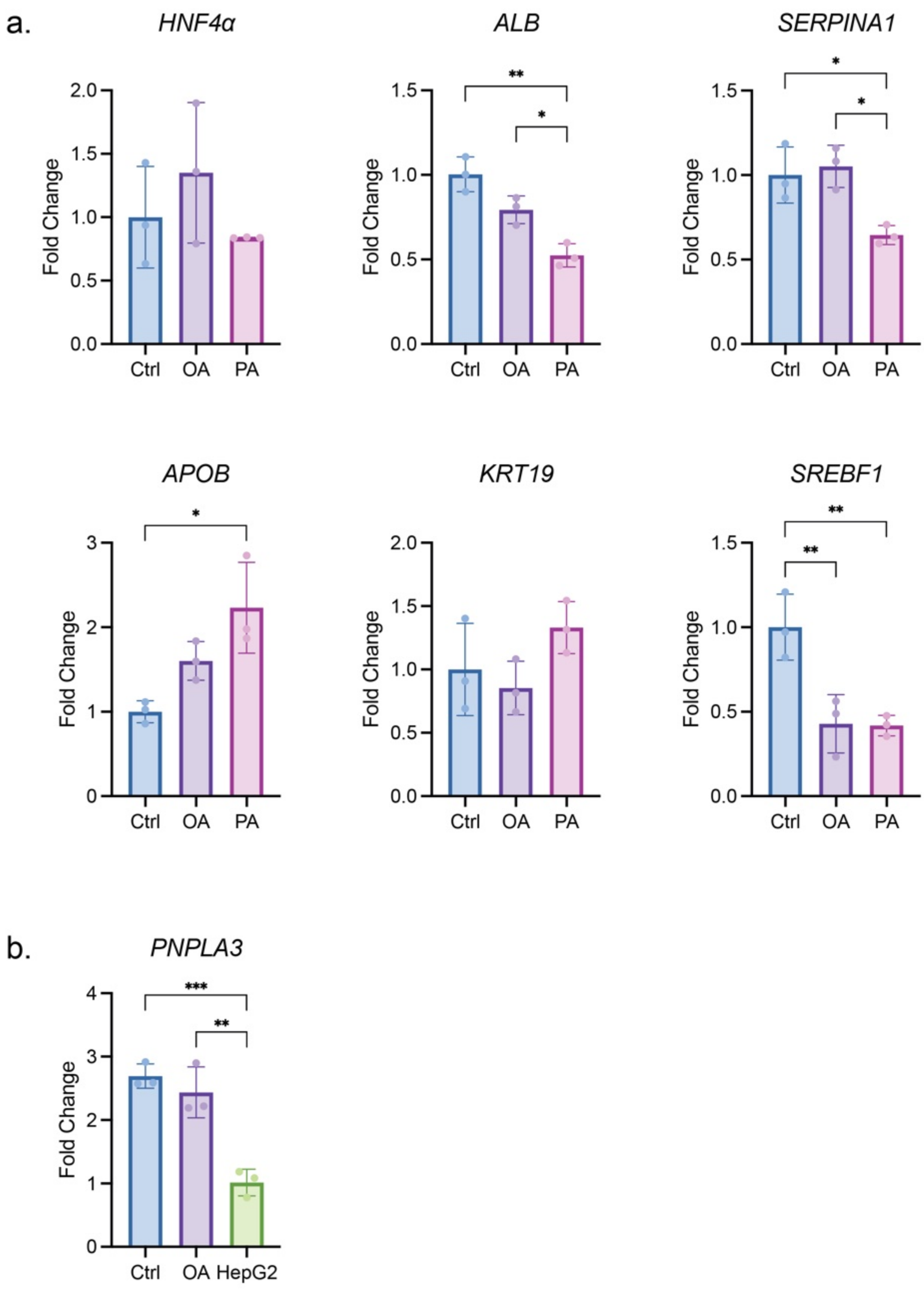
a.
HNF4α
ALB
SERPINA1
APOB
KRT19
SREBF1
Fold Change
Ctrl
OA
PA
b.
PNPLA3
Fold Change
Ctrl
OA
HepG2

Figure S5. Gene expression of MASLD model. **(a)** qRT-PCR results of select hepatic markers comparing the HLC to sHLC. Representative results of 3 biological replicates of differentiation, and each data point entails one technical replicate of the reaction. The p-values for Ctrl vs. OA, Ctrl vs. PA, and OA vs. PA are as follows: 0.5556, 0.8732, 0.3204 for *HNF4α*; 0.0552, 0.0012, and 0.0201 for *ALB*; 0.8696, 0.0300, and 0.0167 for *SERPINA1*; 0.1637, 0.0113, and 0.1435 for *APOB*; 0.7934, 0.3572, and 0.1582 for *KRT19*; 0.0094, 0.0086, and 0.9963 for *SREBF1*. **(b)** qRT-PCR results of PNPLA3 among HLC, OA-induced sHLC, and HepG2. Representative results of 3 biological replicates of differentiation, and each data point entails one technical replicate of the reaction. The p-values for HepG2 vs. Ctrl, HepG2 vs. OA, and Ctrl vs. OA are as follows: 0.0009, 0.0021, 0.5446.

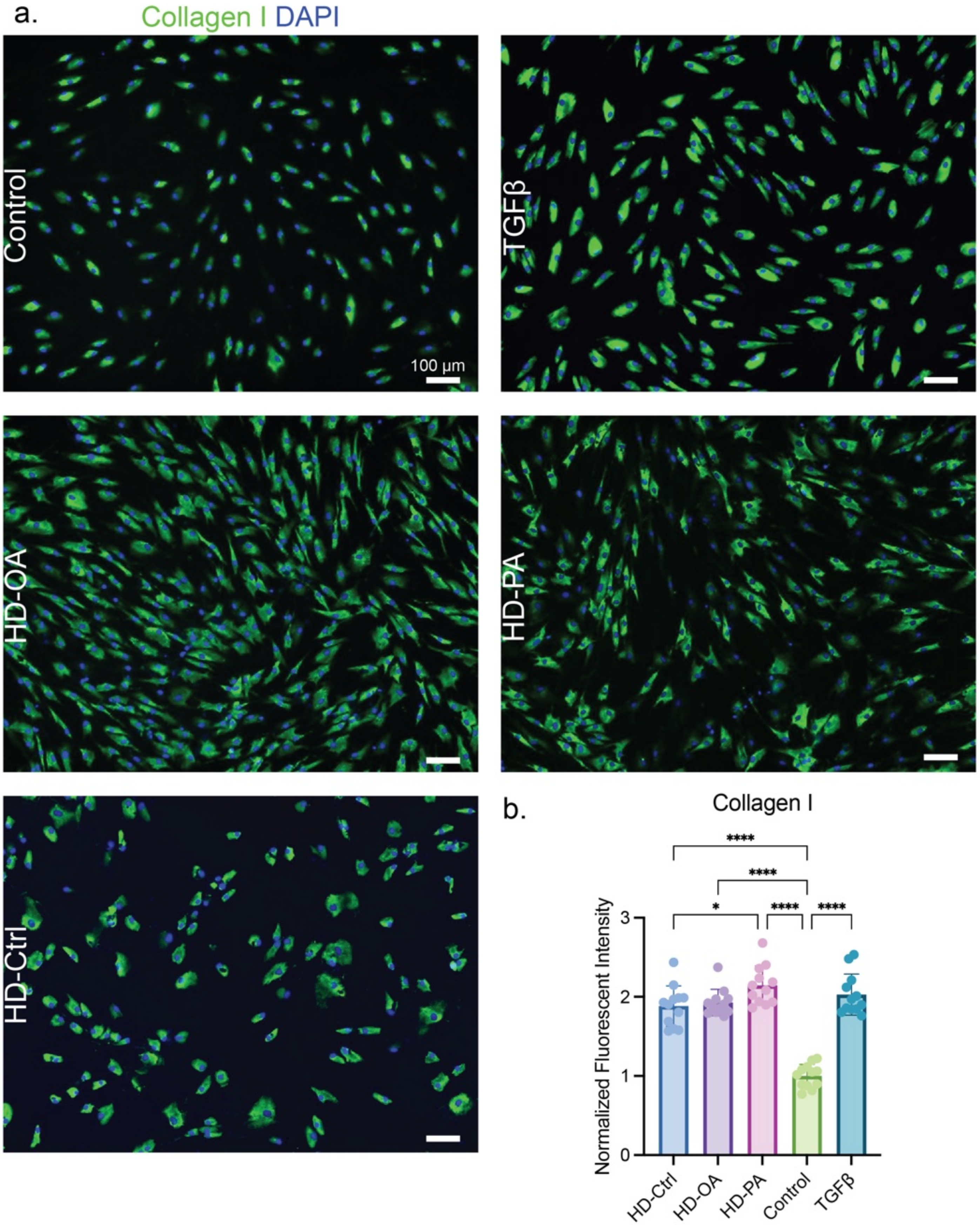
a.
Collagen I DAPI
Control
TGFβ
HD-OA
HD-PA
HD-Ctrl
100 µm
b.
Collagen I
****
****
*
****
****
Normalized Fluorescent Intensity
0
1
2
3
HD-Ctrl
HD-OA
HD-PA
Control
TGFβ

Figure S6. Collagen expression in MASLD model and TGFβ conditions. (a) Staining and **(b)** quantification of collagen 1 production in HDF activated by TGFβ or 2D MASLD model (HD-OA and HD-PA) in comparison to HCM control and 2D HLC Control (HD-Ctrl). Data was generated by normalizing the percentage of collagen 1 coverage per image to the cell count per image. Representative results of 3 biological replicates of differentiation and n = 6 images. The p-values of the Shapiro-Wilk test are 0.6260, 0.0372, 0.3381, 0.0197, and 0.3637 for Control, TGFβ, HD-Ctrl, HD-OA, and HD-PA, respectively. The p-value is < 0.0001 for Control vs. TGFβ, HD-Ctrl, HD-OA, or HD-PA. The p-values are 0.3752 for TGFβ vs. HD-Ctrl, 0.4828 for TGFβ vs. HD-OA, 0.4828 for TGFβ vs. HD-PA, 0.6112 for HD-Ctrl vs. HD-OA, 0.0296 for HD-Ctrl vs. HD-PA, and 0.0921 for HD-OA vs. HD-PA.

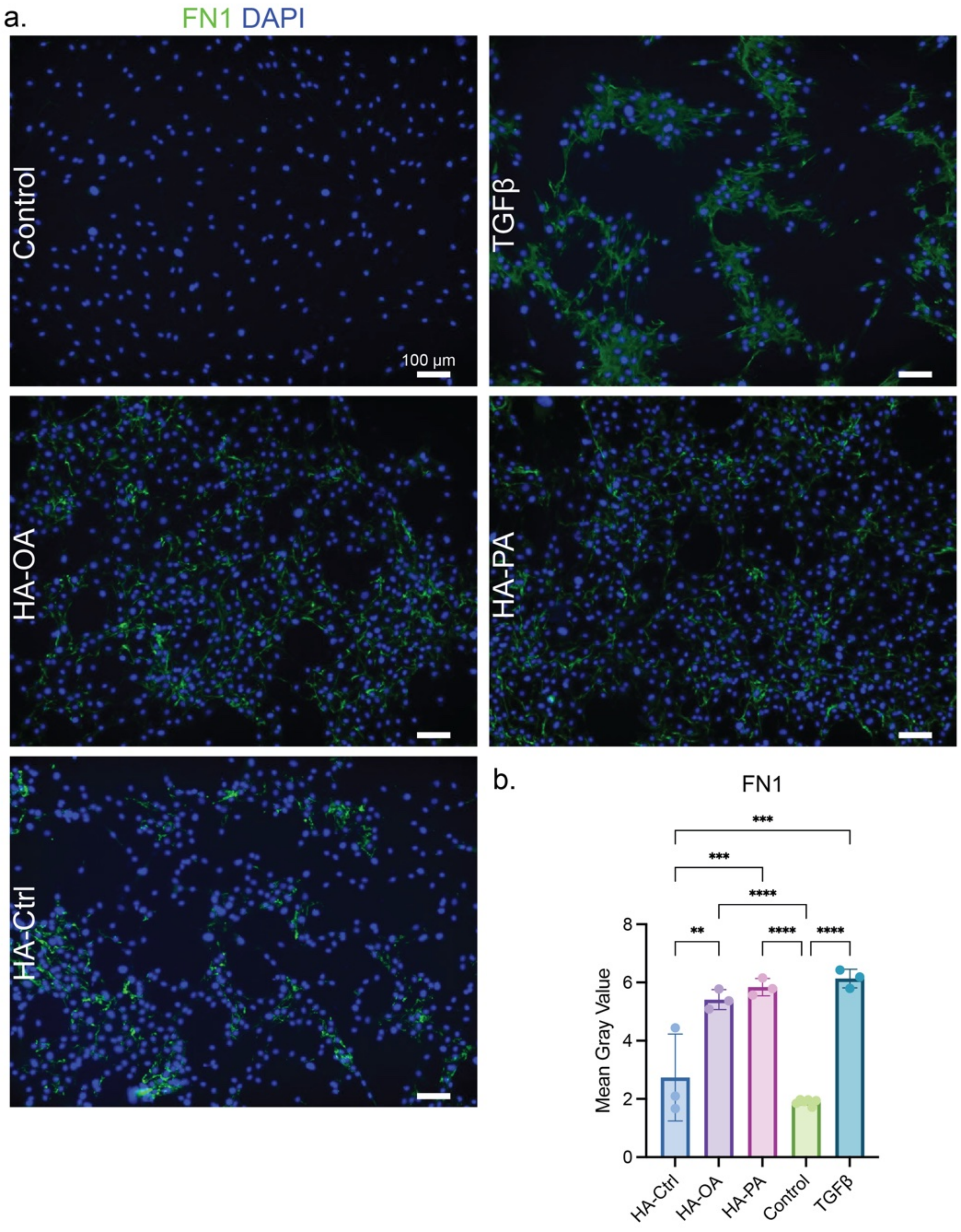
a.
FN1 DAPI
Control
100 µm
TGFβ
HA-OA
HA-PA
HA-Ctrl
b.
FN1
***
***
****
**
****
****
Mean Gray Value
8
6
4
2
0
HA-Ctrl
HA-OA
HA-PA
Control
TGFβ

Figure S7. Fibronectin deposition in TGFβ conditions and 3D MASLD model. **(a)** Staining and **(b)** quantification of fibronectin deposition in HDF activated by TGFβ or 3D MASLD model (HA-OA and HA-PA) in comparison to HCM control and 3D HA Control (HA-Ctrl). Representative results of 3 biological replicates of differentiation, and n = 6 images for HCM control, while n = 3 images for other conditions. The p-values of the Shapiro-Wilk test are 0.1679, 0.7203, 0.2717, 0.7995, and 0.7276 for Control, TGFβ, HA-Ctrl, HA-OA, and HA-PA, respectively. The p-value is < 0.0001 for Control vs. TGFβ, HA-OA, or HA-PA. The p-values are 0.3635 for Control vs. HA-Ctrl, 0.0001 for TGFβ vs. HA-Ctrl, 0.6265 for TGFβ vs. HA-OA, 0.9753 for TGFβ vs. HA-PA, 0.0013 for HA-Ctrl vs. HA-OA, 0.0003 for HA-Ctrl vs. HA-PA, and 0.9142 for HA-OA vs. HA-PA.

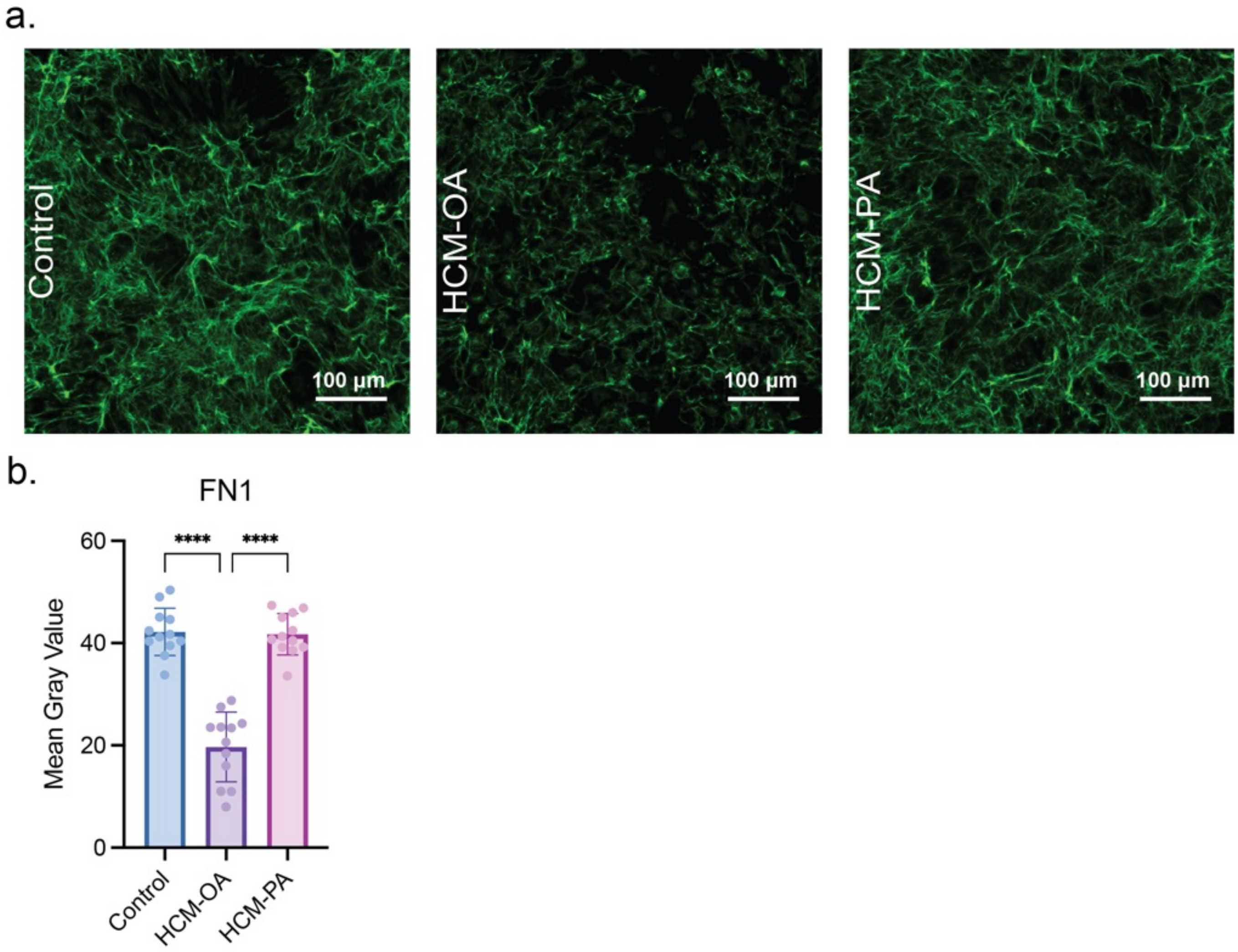

Figure S8. Free fatty acids do not significantly induce fibrotic responses in HDF. (a) Staining and **(b)** quantification of fibronectin deposition in HDF treated by HCM only (Control), HCM with 100 μM OA (HCM-OA), or HCM with 50 μM PA (HCM-PA). Representative results of 3 biological replicates of differentiation, and n = 12 images for each condition. The p-values of the Shapiro-Wilk test are 0.8850, 0.3092, and 0.5698 for Control, HCM-OA, and HCM-PA, respectively. The p-value is < 0.0001 for Control vs. HCM-OA, 0.9772 for Control vs. HCM-PA, and < 0.0001 for HCM-OA vs. HCM-PA.

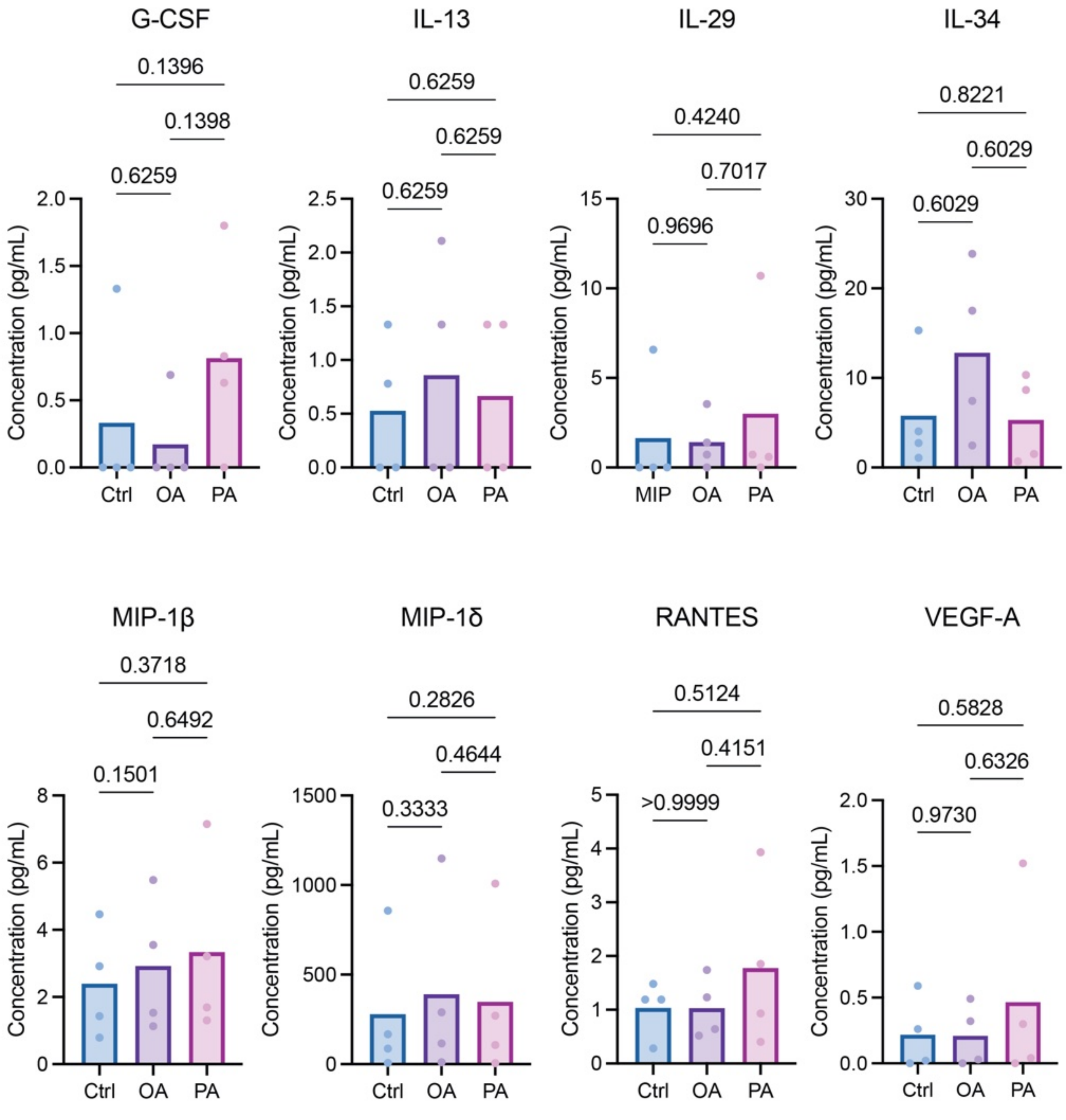

Figure S9. Cytokine Analysis. The concentration of the top 5 cytokines with an increase in relative secretion from either the OA or the PA induced 2D MASLD model (except for MCP-4) in healthy HLC compared to that upon OA or PA induction. Results of 4 biological replicates of differentiation, and each data point entails 2 technical replicates of the reaction. All p-values are labeled above for every comparison between two conditions.

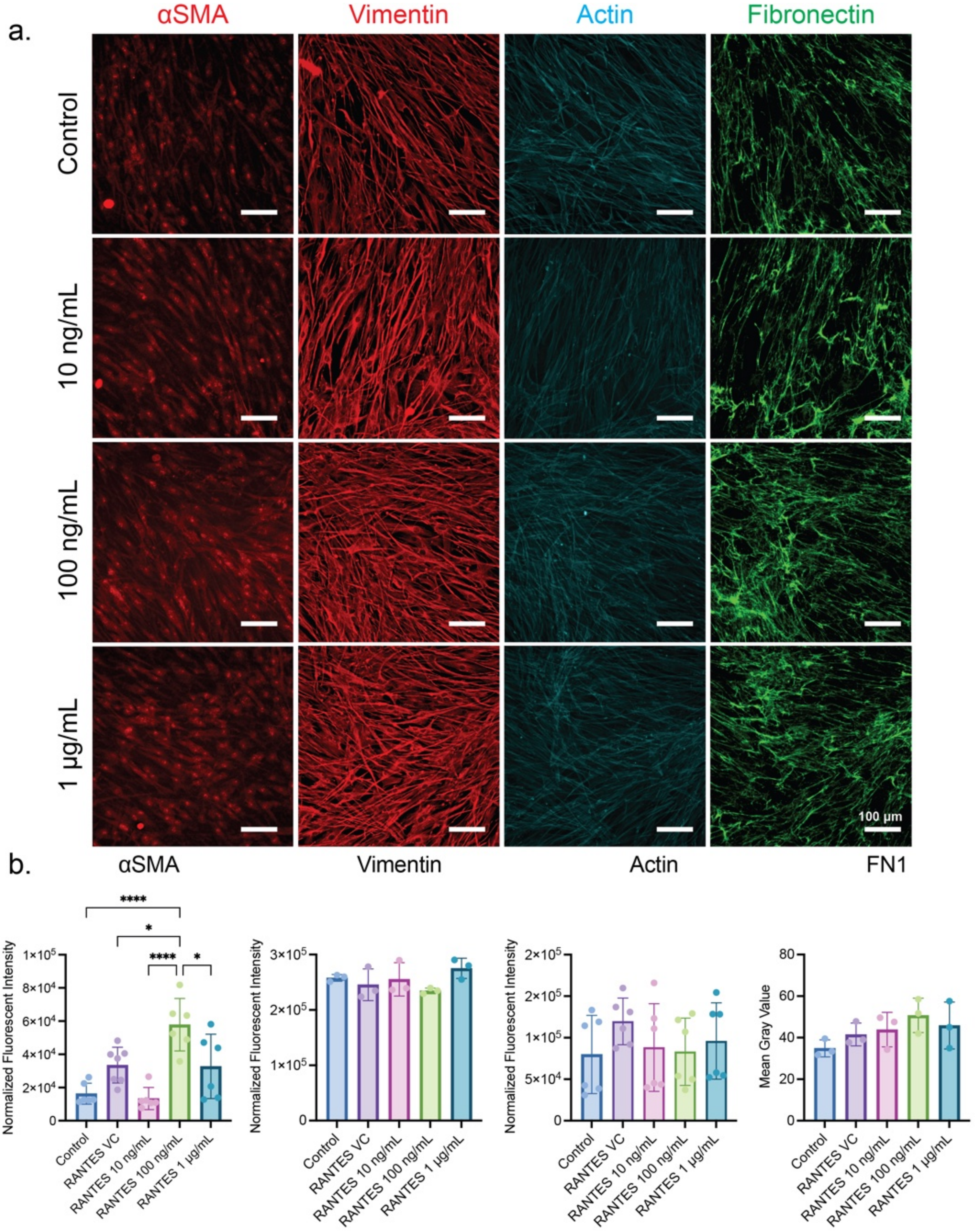
a.
αSMA
Vimentin
Actin
Fibronectin
Control
10 ng/mL
100 ng/mL
1 μg/mL
100 μm
b.
αSMA
Vimentin
Actin
FN1
Normalized Fluorescent Intensity
Mean Gray Value
****
*
****
*
Control
RANTES VC
RANTES 10 ng/mL
RANTES 100 ng/mL
RANTES 1 μg/mL

Figure S10. RANTES does not significantly induce fibrotic responses in HDF. (a) Representative IF images from 3 biological replicates of HDF treated with varying concentrations of RANTES. **(b)** The quantification of IF images for αSMA, vimentin, and actin was achieved by normalizing the total IF intensity to the number of cells, and the quantification for fibronectin was achieved by measuring the fluorescent intensity (mean gray value) per image. Each data point represents one image, with a minimum of 3 technical replicates per biological replicate.
